# Evidence of both molecular cloud and fluid chemistry in Ryugu regolith

**DOI:** 10.1126/sciadv.adp3037

**Published:** 2024-07-24

**Authors:** Maitrayee Bose, Robert A. Root, Yunbin Guan, Jacob Eaton, Axel Wittmann, Thomas Skrmetti, Steven J. Desch

**Affiliations:** ^1^ School of Earth and Space Exploration, Arizona State University, Tempe, AZ 85287, USA.; ^2^ Department of Environmental Science, University of Arizona, Tucson, AZ 85721, USA.; ^3^ Division of Geological and Planetary Sciences, California Institute of Technology, Pasadena, CA 91125, USA.; ^4^ Department of Mathematical Sciences, Arizona State University, Tempe, AZ 85287, USA.; ^5^ Eyring Materials Center, Arizona State University, Tempe, AZ 85287, USA.

## Abstract

The sulfur chemistry of (162173) Ryugu particles can be a powerful tracer of molecular cloud chemistry and small body processes, but it has not been well explored. We report identification of organosulfurs and a sulfate grain in two Ryugu particles, A0070 and A0093. The sulfate grain shows oxygen isotope ratios (δ^17^O = −11.0 ± 4.3 per mil, δ^18^O = −7.8 ± 2.3 per mil) that are akin to silicates in Ryugu but exhibit mass-independent sulfur isotopic fractionation (Δ^33^S = +5 ± 2 per mil). A methionine-like coating on the sulfate grain is isotopically anomalous (δ^15^N = +62 ± 2 per mil). Both the sulfate and organosulfurs can simultaneously form and survive during aqueous alteration within Ryugu’s parent body, under reduced conditions, low temperature, and a pH >7 in the presence of N-rich organic molecules. This work extends the heliocentric zone where anomalous sulfur, formed by selective photodissociation of H_2_S gas in the molecular cloud, is found.

## Teaser

*Hayabusa2* mission returns with Ryugu particles that have preserved sulfate and organosufur compounds.

## INTRODUCTION

Compared to all the extraterrestrial materials available on Earth, particles from the carbonaceous chondrite (CC) asteroids (162173) Ryugu and (101955) Bennu are the least terrestrially contaminated samples from small-body regolith of the outer solar system. Bulk and microanalyses of Ryugu particles have revealed ubiquitous phyllosilicate minerals including smectite and serpentinite (*1*–*3*), as well as carbonates and magnetite minerals produced by pervasive aqueous activity at temperatures below 300°C (*2*), and the particles exhibit substantially more porosity than in meteorites (*4*–*5*). Organic matter also occurs in Ryugu particles, as diffuse micrometer to submicrometer domains with aromatic-poor and carboxylic-rich compositions (*6*). The bulk oxygen isotope ratios of Ryugu particles indicate that Ryugu is similar to CI (Ivuna-like) CC meteorites with both ^16^O-rich (akin to calcium-, aluminum-rich inclusions) and ^16^O-poor (chondrule-like) compositions (*1*).

The particles retrieved from asteroid Ryugu allow us to investigate the chemical evolution of the biocritical element sulfur in a low-temperature, water-rich asteroid body. Although sulfur primarily occur as inorganic sulfides, an inventory of sulfur-bearing organic compounds in Ryugu is currently unknown. An extractable subset was recently reported on, from in the salt-, carbonate-, and phyllosilicate-bearing fractions of two particles from Ryugu (A0106 and C0107) (*7*). The salt fraction extracts contain anionic soluble sulfur-bearing species such as polythionic acids, alkylsulfonates, and hydroxyalkylsulfonates (*7*). Thiosulfate was detected in the water-soluble extracts (*7*). The δ^34^S [defined as the ratio of ^34^S/^32^S to a terrestrial reference ratio and expressed in per mil (‰)] values in both surface and subsurface particles (bulk) were similar (−1.1 ± 1.6‰ and −3.0 ± 2.3‰) within errors (*7*). The goal of this work is to analyze Ryugu samples to determine the formation pathway and chemical microenvironment of sulfur-bearing organic matter and inorganic minerals, and the astrophysical origins of the isotopically anomalous sulfur.

Pyrrhotite [Fe_(1−*x*)_S, *x* = 0 to 0.2] and pentlandite [(Fe,Ni)_9_S_8_] with oxidation state S^2−^ are the dominant sulfide minerals in the CM (Mighei-like) CCs [e.g., (*8*)]. Hydrous tochilinite/cronstedtite (6(Fe_0.9_S)·5[(Mg,Fe)(OH)_2_]/Fe^2+^2Fe^3+^[(Si,Fe^3+^)_2_O_5_](OH)_4_) intergrowths with mackinawite (Fe^2+^S^2−^) are observed as alteration products in CM chondrite matrices (*9*–*10*). Other sulfides, such as greigite (Fe_3_S_4_), keilite (FeMgS), oldhamite (CaS), and sodium sulfide (Na_2_S), have also been identified in chondritic meteorites [e.g., (*11*–*13*)]. The CM chondrites contain gypsum (CaSO_4._ 2H_2_O; S^6+^) while the CI1 (Ivuna-like) CCs display veins of sodium sulfate, calcium sulfate, and magnesium sulfate argued to have terrestrial origins [e.g., (*14*–*15*)]. In addition to the inorganic sulfur minerals, sulfur-bearing organic compounds have been identified in water- or solvent-soluble extractions, e.g., methyl, ethyl, isopropyl, and *n*-propyl sulfonic acids from Murchison (*16*). Heteroatom- and sulfur-containing aromatic compounds, e.g., thiophenols, benzothiophenes, and alkylated thiophenes, were detected in extracted insoluble organic matter [e.g., (*17*–*20*)].

Here, we present multimodal results from the analysis of sulfur oxidation states and isotopes in two Ryugu particles A0070 and A0093 from chamber A (both particles were collected during the first touchdown of the Hayabusa2 mission). We found an organosulfur [thiol (R-SH) and thioester (R-S-R), generally referred to thiol hereafter] and sulfate (SO_4_)–bearing composite grain in Ryugu particle A0070 that shows a mass-independent sulfur isotopic fractionation. Its oxygen isotope ratios are similar to those observed in anhydrous silicate minerals in Ryugu samples. We identified sulfur in various oxidation states, including thiols and sulfoxide. The diverse sulfur speciation can be used to place useful geochemical constraints of the Ryugu parent body and evaluate the redox state of its microenvironments. The occurrence of the sulfate grain and organosulfur species clearly indicates that both primordial molecular cloud material and its alteration products from aqueous processing are well preserved in Ryugu. We acquired similar data from two other CC meteorites, CM2 Murchison and CR2 Graves Nunataks (GRA) 95229 to compare the distribution of the sulfur oxidation states. The total sulfur abundance in Ryugu particles (3.3 to 5.5 wt %) (*6*) is higher compared to that reported in Murchison and GRA 95229 (1.2 to 3.3 wt %) (*21*). Data and discussion on the meteorites are in the Supplementary Materials.

## RESULTS

**Fig. 1.**
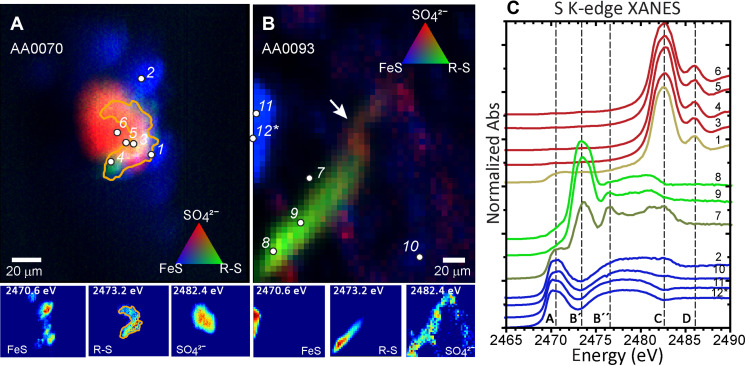
Stacked multiple-energy x-ray fluorescence maps collected across the sulfur absorption edge reveals two thiol-rich regions identified in Ryugu particles A0070 and A0093. (**A**) The thiol (in green) forms a partial rim, about 10 μm wide around a calcium sulfate grain (in red). The rim is surrounded by smaller monosulfide grains (in blue). (**B**) The thiol forms a (60 μm by 20 μm) filamentous structure that fades into a methionine sulfoxide and then sulfate-rich region (shown at arrow). This exists in a phyllosilicate-rich region in close proximity to a monosulfide grain. (**C**) Sulfur K-edge XANES were collected at spots of interest [points 1 to 12 shown in (C)], and normalized spectra are shown. Vertical dashed lines in the plots of spectra show the following: (A) inorganic monosulfides (Fe_1−*x*_S) pyrrhotite/troilite; (B′) cysteine as a representative thiol (R-SH); (B″) methionine sulfoxide (R-(SO)-R; (C) characteristic S^6+^ sulfate (SO_4_^2−^); (D) secondary peak characteristic of Ca-sulfate (CaSO_4_) such as gypsum. XANES fits are shown in table S1.

We scanned a total of 15 and 8 areas in particles A0070 and A0093, respectively via synchrotron micro–x-ray fluorescence (μXRF) imaging and micro–x-ray absorption near-edge structure (μXANES) spectroscopy. Each area was typically between 300 × 300 μm^2^ and 500 × 500 μm^2^ in size. Most of the monosulfides in the Ryugu particles, identified by characteristic μXANES spectra, occur in the fine-grained matrix and are of mixed composition; the presence of metals (possibly iron in different abundances) is indicated by the clear doublet sulfide spectral peak (2470.5 ± 0.5 eV). We found a 40 μm × 40 μm sulfate grain (mapped at its characteristic peak energy of 2482.5 eV) in particle A0070, with a partial coating of thiol (mapped at 2473.2 eV) proximal to inorganic sulfides (<2472 eV) (Fig. 1A); referred to as A0070_TSul#1 hereafter. A second thiol-rich domain was found within the particle A0093 (called A0093_TSul#2), which also shows evidence of sulfate (Fig. 1B). A portion of the thiol domain in A0093_TSul#2 shows occurrence of sulfoxide (2476.3 eV) such as methionine sulfoxide (2476.2 eV; Fig. 1B). Thiols, containing the ─SH functional group, are the simplest sulfur-bearing organic compounds, and this is the first in situ identification of thiol in Ryugu particles. Detections of nonterrestrial thiols in meteorites are quite rare, although they may occur (see the Supplementary Materials). Nevertheless, their detection in Ryugu highlights their presence in hydrated asteroids and enhances our current understanding of the origins of organosulfur-bearing phases in the Ryugu parent body.

The occurrence and spectral overlap of the elemental sulfur (2472.0 eV) in the vicinity of the thiol in the sulfur XANES spectra can make the assessment of thiol contents difficult (*19*–*20*). However, the identification of thiol in Ryugu particles is unambiguous, which shows a clear peak at (2473.3 eV) corresponding to the thiol feature (Fig. 1C). We do not observe a dominant thiol peak in the spectra of CC meteorites Murchison and GRA 95229 but we do observe a shoulder on the elemental sulfur peak (see the Supplementary Materials for a comprehensive discussion of the meteoritic data; table S1). An asymmetric peak shape or shoulder above 2472.0 eV (location of the elemental sulfur peak) can be attributed to the presence of thiol (fig. S6). These asymmetric peaks occur in GRA 95229, from which we surmise that the thiol phases are likely destroyed in CC meteorites quite promptly after their fall or during sample processing to extract insoluble organic matter.

**Fig. 2.**
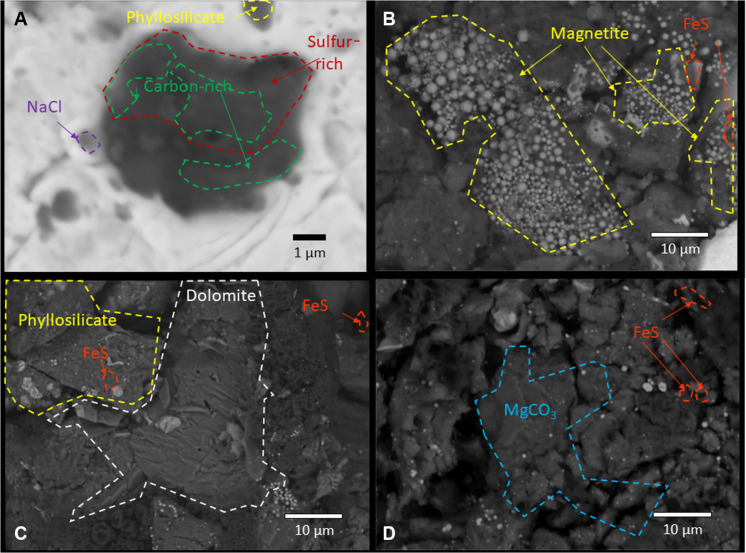
Backscattered electron images of Ryugu particle A0070 showing the variable mineralogy. Area labels are as follows: (**A**) Ca-thiosulfate A0070#1 (Fig. 1). (**B**) Fe sulfides and magnetite framboids. (**C** and **D**) Ca- and Ca-, Mg-bearing carbonate minerals.

Backscattered electron images and elemental maps were acquired with an electron microprobe for several areas on the Ryugu particle A0070 to characterize the general mineralogy of the particles and locate the Ca-sulfate grain for isotopic analysis. The P-bearing hotspots were small and generally associated with Ca, most likely apatite, which is generally argued to be a hydrothermal alteration product in CM chondrites. Abundant FeS grains were present, which may originate from a cooling gas in the protosolar nebula and their subsequent incorporation into the Ryugu protolith. In addition, the Ryugu particle contained several areas with framboidal magnetite, abundant phyllosilicates, sulfides, and calcites (Fig. 2). The Ca-thiosulfate grain A0070-TSul#1 was identified (Fig. 2A) and shows presence of carbon and sulfur. The same area shows presence of smaller grains of halite (~0.5 μm), calcite (~0.5 μm × 1 μm), and phyllosilicate minerals (~1 μm). Abundant <100-μm-sized dolomite grains and rarer, but larger breunnerite grains have been identified in Ryugu particles.

Ryugu particle A0070-TSul#1 exhibits a sulfur isotopic composition of δ^33^S = −0.1 ± 1.6‰, δ^34^S = +9.8 ± 0.9‰, Δ^33^S = +5 ± 2‰ (1σ). The oxygen isotope compositions of the Ryugu Ca-thiosulfate grain A0070-TSul#1 are similar to those of isolated Mg-rich chondrule-derived olivine and pyroxene minerals (*22*–*24*) (Fig. 2B), lying on the Carbonaceous Chondrite Anhydrous Mineral (CCAM) line. Both A0070-TSul#1 (δ^13^C = −99 ± 1‰) and the methylsulfonic acid from Murchison (*16*) exhibit carbon isotopic anomalies, with the thiol-rich coating on A0070 sulfate showing ^15^N-rich signatures (δ^15^N = +62.3 ± 2‰).

## DISCUSSION

**Fig. 3.**
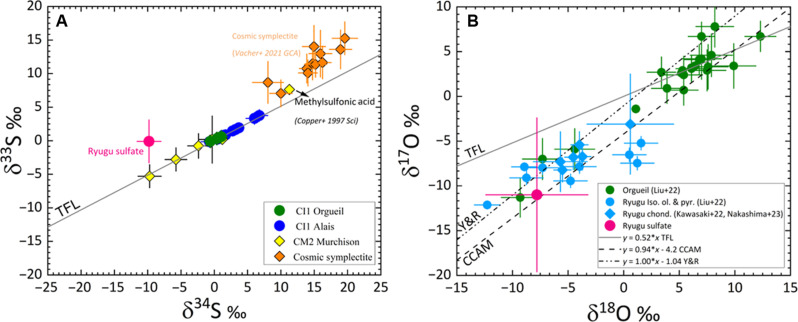
Oxygen and sulfur isotopic compositions of the Ca-thiosulfate grain in A0070. (**A**) Three-sulfur isotope plot showing the sulfur isotopic composition of sulfides in several CC meteorites, cosmic symplecties (*25*), methylsulfonic acid (*16*), and the thiol- and sulfate-bearing composite grain identified in Ryugu particles A0070 with a Δ^33^S = 5 ± 2‰ (1σ). A normal sulfur isotopic fractionation process means that a change in mass of 2 amu (^34^S -^32^S) for δ^34^S is accompanied by a 1 amu for δ^33^S. This results in an observed factor of 2 for a mass-dependent fractionation owing to chemical and physical processes. Any deviation from it is considered “isotopically anomalous” or mass independent in nature, which is the case for the Ca-thiosulfate grain in the Ryugu particle A0070. (**B**) The O isotopic composition of Ryugu Ca-thiosulfate grain A0070-Sul is consistent with that measured in a few points in Orgueil as well as isolated olivines and pyroxenes and chondrule fragments in Ryugu (*22*–*24*) The Terrestrial Fractionation Line (TFL), Young and Russell (Y&R) line, and CCAM line are also shown. The TFL is derived from measurements of terrestrial samples falls along a line with slopes (~0.5). The CCAM line is derived from analyses of refractory inclusions in CV3 CC Allende has a slope of 0.94. It is used for assessing the magnitude by which planetary and asteroidal materials depart from the three-isotope distribution of Earth’s oxygen reservoir. Young and Russell (*61*) have proposed that a line of exactly slope 1 defines the primordial variation in oxygen isotopes in the protosolar disk. This primitive oxygen isotope composition was subsequently modified by later parent body processes to form the CCAM line. Data are in table S11.

Ryugu particle A0070-TSul#1, like methylsulfonic acid (CH_4_O_3_S) in CM2 Murchison (*16*) and cosmic symplectites (COS) in ungrouped CC Acfer 094 (*25*) exhibit a sulfur isotopic anomaly Δ^33^S = δ^33^S–1000 × [(1 + δ^34^S/1000)^0.515^–1] > 0‰, indicating mass-independent sulfur isotopic fractionation (Fig. 3A). For comparison, sulfides in Orgueil and other CM chondrites show Δ^33^S = 0‰, indicating only mass-dependent sulfur isotopic fractionation (Fig. 3A) (*26*, *27*). Ultraviolet photolysis of SO_2_ under reducing conditions (pO_2_ < 10^−6^ atm) could produce isotopically fractionated S^0^ and SO_4_^2−^ (*28*, *29*), but it is not clear whether the required conditions (e.g., SO_2_ gas) would exist, or whether the observed fractionation (Δ^33^S = 5 ± 2‰) would be produced. In contrast, the mass-independent fractionation of sulfur isotopes in COS in Acfer 094 were attributed (*25*) to isotopically selective photodissociation of H_2_S vapor in warm (*T* > 70 K) molecular gas, which produces isotopically heavy (Δ^33^S, Δ^36^S > 0‰) S^0^ that is accreted into ices, ultimately accreted by chondrite and Ryugu parent bodies and mobilized by aqueous alteration. On the basis of the Δ^33^S/Δ^36^S ratio in COS, the study in (*25*) attributed the radiation source to nearby massive OB stars irradiating molecular cloud gas, rather than Lyα irradiation from the early Sun irradiating disk gas. Although we do not have Δ^36^S data to perform this same diagnostic, we note that our measured Δ^33^S = 5 ± 2‰ is an excellent match to that of COS, Δ^33^S = 3.84 ± 0.72‰ (*25*), and we infer that Ryugu accreted the same isotopically heavy reservoir of sulfur as Acfer 094. Such irradiation should have occurred within 1.8 million years after time 0 (formation of calcium-, aluminum-rich inclusions) (*30*).

Our results extend the zone across which the isotopically anomalous sulfur reservoir existed. The meteorites in which it was previously found (Murchison, Acfer 094) are chondrule-bearing CCs that presumably formed immediately outside the then-orbit of Jupiter, at about 3 to 4 astronomical units (au) (*31*). Ryugu, lacking chondrules, appears more akin to CI chondrites [e.g., (*1*–*3*)] that seem to have formed beyond 15 au from the Sun (*31*). Where Ryugu formed, the flux of Lyα from the early Sun would be 25 times weaker than where Murchison formed, which supports models in which photodissociation occurred in the molecular cloud. Such an origin is further supported by the widespread radial distribution of the isotopically anomalous sulfur reservoir and the association with large δ^15^N anomalies, attributable to molecular cloud material [e.g., (*32*)].

The oxygen in the Ca-thiosulfate likely derives from the water with which the isotopically anomalous elemental S^0^ reacted within accreted ices. This water probably derives from the same ^16^O-poor water reservoir sampled by the COS in Acfer 094 (*33*) but mixed with more isotopically normal water that is equilibrated with silicates in the Ryugu parent body. Experiments have shown that forsteritic olivine grains <50 μm in size would be largely altered and form phyllosilicates in approximately 10 years at 25°C due to chemical exchange with water (*34*). However, aqueous processing is not expected to have altered the oxygen isotope of the sulfate after its formation. The diffusion timescales of oxygen in known materials are too slow, especially at lower temperatures (*35*) because oxygen bonds with cations efficiently and has difficulty migrating within a crystalline structure. Sulfate, once produced, is inhibited from oxygen isotope exchange with water unless temperature substantially exceeds 100°C or the pH falls below a value of 2 (*36*–*37*). Therefore, akin to the anhydrous silicates in Ryugu, A0070-TSul#1 precursors were incorporated into Ryugu protolith and survived fluid activity without oxygen isotopic exchange in the asteroid parent body.

Rare domains with ^15^N excesses have been reported in Ryugu by Ito *et al.* (*38*) (up to 550‰ in δ^15^N with an average of 260‰) and Nakamura *et al*. (*39*) (δ^15^N = 312‰). The bulk δ^15^N values of Ryugu particles are an order of magnitude lower [δ^15^N = 35 to 45‰; (*3*, *38*–*41*)] and the N-bearing organic coating described here is slightly more enriched than the bulk. Although the most extreme isotopic anomalies reported in Ryugu likely require an origin at extremely low temperatures, either in the interstellar medium or in the far outer Solar System (*42*, *43*), the bulk of the organic precursor materials is a result of a stochastic mixing or homogenization process [e.g., (*41*)]. These processes could have occurred in the protoplanetary disk, before accretion into small bodies and/or by hydrothermal alteration in the interior of these bodies. Because Ryugu is anomalous in its N isotopes, then it is possible that the thiol-rich domains formed within the Ryugu interior with preexisting ^15^N-rich organic matter and that thiols are another carrier phase of N-bearing materials. NH-bearing phases, e.g., NH_4_ phyllosilicates, hydrated salts, or organics have been confirmed in Ryugu particles with the 3.1-μm infrared feature (*44*). These species reflect preserved NH-bearing ices during accretion, while the thiol coatings around A0070-Sul are a result of phases that formed in situ in the Ryugu parent body.

**Fig. 4.**
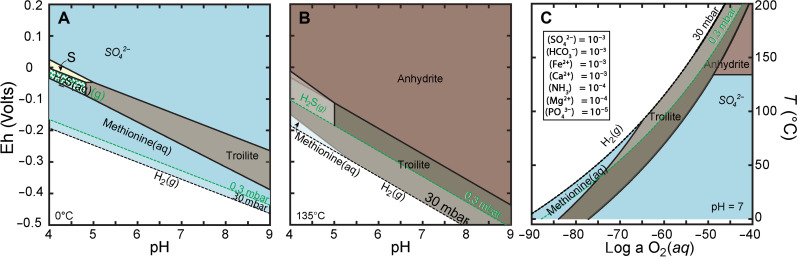
Eh-pH (Pourbaix) diagram for stable sulfur compounds in Ryugu. Sulfur species stability boundaries (*E*_h_-pH diagrams) calculated as a function of redox potential and pH at 0°C (273 K) (**A**) and 135°C (408 K) (**B**), and S species and as a function of temperature from 0° to 200°C (273 to 473 K) and log O_2_ activity from −90 to −40 M at pH = 7 (**C**). Solid black lines define sulfur phase and species boundaries at 30 mbar, and green lines show the same boundaries at 0.3 mbar. Blue fields are aqueous; the stability field of troilite (Fe_1−*x*_S) is tan, that of anhydrite (CaSO_4_) is red, and that of gaseous hydrogen sulfide (H_2_S) is white. Aqueous H_2_S coexists with gaseous H_2_S in regions marked with green hashed lines. Black dashed lines show the stability boundary of water and H_2(_*g*) at 30 mbar, and stippled green lines show the H_2(_*g*)- H_2_O boundary shifts from 30 to 0.3 mbar. Model input solution composition data [shown as inset in (C)] are assumed from (*3*) and constrained by known C-type asteroids; temperature limits are assumed to be < ~150°C for Ryugu particles (*6*, *38*). Activities of species yet unreported were assigned a log activity of 10^−3^, an assumption consistent with known constraints from C-type asteroids. Pyrite precipitation was suppressed in the model. The broad predominance field of stable methionine across the wide pH range at all low-O_2_ conditions demonstrates thiol species in close association with sulfate and sulfides are thermodynamically permissible.

Our work suggests that formation of organosulfur compounds in a low-temperature asteroidal environment is inevitable, and an essential part of small-body aqueous chemistry. Sulfur itself is ubiquitous in meteorites and in asteroid Ryugu and is present in sulfur-bearing minerals (e.g., pyrrhotite and pentlandite). These sulfur-bearing mineral assemblages can function as redox buffers, surface catalysts, or both. In addition, synthesis of nitrogen-bearing thiols from abiotic precursors requires nitrogen as organic matter and carbon sources such as CO_2_ and CO as the most likely starting materials. These starting materials should lead to the formation of methanethiol, ethanethiol and other longer-chained thiols. To understand this process and place constraints on the characteristic of Ryugu’s fluid composition and character, we carried out thermodynamic modeling (Geochemist Workbench) based on reported Ryugu chemical and mineralogic makeup. We assume mineral components such as Fe, Mg, Ca, as well as CO_2_, and NH_3_ were available in variable abundances and that compositional changes occur at short timescales, though no kinetic modeling was attempted. Thermodynamic modeling shows that methionine is stable over a wide geochemical stability field or “space” in the presence of aqueous carbon, at low-O_2_ activity (i.e., reducing conditions; e.g., 10^−80^ M), lower temperatures (0° to 100°C, model limited to ≥0°C), and moderate to higher pressures (> 10^−7^ bar) (Fig. 4). Figure 4A shows the stability of CaSO_4_(*aq*), troilite and methionine at 0°C. Here, thermodynamically stable anhydrite, the dehydrated calcium sulfate endmember, is modeled, but hydrated polymorphs gypsum or bassanite (CaSO_4_·0.5H_2_O) cannot be ruled out as the XANES identified S^6+^ sulfate species. These three species are predicted to coexist at a pH ≥ 7, within the expected pH range derived from Fe^2+^/Fe^3+^ in Ryugu minerals (*45*). Elemental sulfur and H_2_S (temperature- and pressure-controlled gas and liquid phase) are stable only at acidic conditions (Fig. 4A). Increasing the pressure does not substantially change the sulfur speciation stability fields, other than shifting the phase boundary of liquid water and H_2_(*g*) (Fig. 4, A to C). Formation of anhydrite and its stability with troilite occur only at *T* > 135°C (Fig. 4B), when Mg activity is lower than Ca activity [(Mg) < 10^−3^ < (Ca)]. At higher temperature, the stability field of methionine is drastically reduced to pH ≤ ~5. Modeling the system at pressure from 0.3 to 30 mbar does not have a substantial effect on methionine stability. Comparing Fig. 4A and Fig. 4B, we surmise that the formation of the thiol coating on the sulfate grain occurred in the low-temperature regime and at pH ≥ 7, and under reduced conditions with a Eh value of about −0.3 V. Figure 4C shows that the aqueous sulfate and anhydrite stability boundary (at 135°C) is temperature controlled and independent of redox. However, the methionine stability field is dependent on both temperature and reducing conditions (Fig. 4C). Thermodynamic modeling indicates, we can place an upper limit of <92°C on the temperature experienced by Ryugu particle A0070. Modeling at high pH, this upper limit in temperature decreases to about 50°C at pH 9. Thus, we predict that the sulfate and thiols formed under relatively low temperature in alkaline, reducing fluids, and their rarity highlights the spatial variability of the fluid composition.

Although the physicochemical conditions in which the thiols in Ryugu particles A0070 and A0093 formed are well constrained by the thermodynamic models, the timing of their formation is unknown. The two possibilities include radiogenic heating very soon after the Ryugu parent body had formed and due to impact heating in the wake of the catastrophic collision that caused Ryugu’s breakup. We prefer the former case, where radiogenic heating primarily by ^26^Al would melt the accreted ices and allow chemical reactions to occur. Events that initiate carbonate formation, dissolution of primary silicate minerals, and formation of sulfate and thiols can occur at the same time, albeit under variable environmental conditions. For the Ca-thiosulfate to form and retain the anomalous sulfur isotopic composition (akin to COS), we are assuming two different environments. Sulfates presumably form near the surface of the parent body, where temperatures are low. However, hydrothermal activity in the interior of Ryugu parent asteroid at much higher temperatures and for a much longer duration would introduce water that is equilibrated with silicates. In this scenario, the water would have the same oxygen isotopic signature as the silicates. Where those two types of fluids (high Δ^33^S surface ice and equilibrated presumably S-free water from depth) mixed, you would have water that equilibrated with silicates for oxygen but retaining the high Δ^33^S signature. Our geochemical modeling shows the temperature of grains with preserved thiols could not have exceeded ~90°C (Fig. 4C), unlike previous constraints from Ryugu regolith particles that set an upper limit to the temperature of <150°C (*6*, *44*).

In summary, the mechanism for the formation of the Ca-thiosulfate grain A0070_Sul#1 in Ryugu is a series of steps, starting with the creation of an isotopically anomalous S^0^ reservoir in the molecular cloud, due to irradiation by nearby massive stars, same as the COS (*25*). This elemental sulfur would have been incorporated into ices accreted by the Ryugu parent body and mobilized to participate in aqueous chemistry. The O isotopes of the sulfate and isolated silicates are consistent with equilibration on Ryugu. The fluid, containing^15^N-rich ammonia or organics, would have interacted with the anomalous sulfur reservoir forming the sulfate grain A0070_TSul#1, and aqueous methionine would have precipitated as a rim on the sulfate near the end of the aqueous alteration period on Ryugu, or during the lithification process. The presence of organosulfurs in Ryugu particles suggests that Ryugu has not seen extensive oxidative processes throughout its history and that parts of Ryugu are much less oxidized than Orgueil (*7*, *46*). The absence of fresh fluids and presence of H_2_S(*g*) help to preserve organosulfurs against further oxidation or destruction.

## MATERIALS AND METHODS

### Ryugu sample preparation

The opening of the sample container from chamber A (from the first touchdown) from the return mission of Japanese spacecraft *Hayabusa2* with the Asteroid 162173 Ryugu particles was done within an anaerobic chamber (COY Labs, MI USA) with a H_2_ (2.6%) and N_2_ (97.4%) environment at the Stanford Linear Accelerator Center (SLAC) National Accelerator Laboratory. The anaerobic chamber was thoroughly cleaned and pumped down overnight before mounting of the Ryugu samples. All tools and materials that were introduced into the chamber were cleaned before and after being introduced into the anaerobic chamber. This approach enabled the characterization of the sulfur inventory in the two Ryugu particles without being influenced by contamination or oxidation. See the Supplementary Materials for a detailed discussion of mounting and subsequent XANES and XRF measurements of references and Ryugu materials.

### XANES and XRF methodology and data collection

XANES is highly sensitive to oxidation state and coordination environment of the absorbing atom, while XRF mapping allows for a unique element-specific visualization at the sample surface. Sulfur speciation was analyzed with μXRF and μXANES spectroscopy on Ryugu particles A0070 and A0093, and meteorites Murchison and GRA 95229 thin sections. Speciation of the Ryugu particles were investigated with micro-focused XANES spectroscopy at the S Kα edge (2742.04 eV) at beam line 14–3 at the Stanford Synchrotron Radiation Lightsource (SSRL) at SLAC, a National Laboratory user facility operated by the Department of Energy. The x-ray beam was operated at 3 GeV and 500 mA, with a double-crystal monochromator (Si [111] crystal, ɸ = 90) used to tune the incident energy on the sample. A Kirkpatrick-Baez mirror system was used to achieve a micro-focused beam (<5-μm spot size). Energy was calibrated using the centroid of the first peak of sodium thiosulfate, assigned to 2472.02 eV. Energy resolution (Δ*E*/*E*) at the sulfur edge is 10^−4^.

Several coarse scale μXRF elemental maps (500 μm by 500 μm, 10- to 20-μm steps) were collected at 2500 eV to locate Al, Si, P, and S from both Ryugu particles A0070 and A0093 to survey the heterogeneity of the particles. This enabled us to create sulfur isopleth (heat) maps and locate sulfur-rich hotspots and to determine its association with other low-Z elements probed at 2500 eV (i.e., Al, Si, P, and Mg). Subsequently, finer-scale areas were mapped at 3- and 1-μm-scale resolutions at multiple-energy (ME-μXRF) maps across the S Kα absorption edge at 2471.1, 2472.6, 2473.5, 2476.4, 2480.1, 2482.3, 2482.8, and 2500.0 eV to exploit the energy differences of the expected S species (*20*, *47*). μXANES spectra were acquired from regions of interest within the mapped areas with the same beam spot size to confirm the speciation of the sulfur-bearing domains. μXRF maps were collected with the sample at 45° from the incident beam and detector, this correction was applied to ensure proper scaling in all figures.

The measured intensity in each pixel is a function of incident energy, fluorescence yield, elemental abundance, and matrix-dependent sampling depth. Collecting and stacking multiple energy maps across the sulfur-edge reveals spatial differences in sulfur oxidation state and speciation. To produce sulfur speciation maps, an energy versus normalized fluorescence intensity matrix was generated using known standard mineral references (*48*). Applying the matrix to the stacked maps of meteorite samples gives a pixel-by-pixel map sufficient for distinction of sulfur species. To validate the maps, images were processed by linear combination and k-means cluster analysis to determine distinct chemical component regions, and μXANES spectra were collected from those points of interest (ca. 50 in each; 3 μm^2^). Mapping at the sulfur edge also fluoresces lower Z elements which can be isolated with an energy discriminating detector (4-element silicon drift Vortex, Hitachi) that allows spatial detection of phosphorus (2014 eV), silicon (1740 eV), aluminum (1487 eV), and magnesium (1254 eV) (vacuum techniques are required to be <1250 eV).

### XANES data processing

XANES spectra were collected over the energy range of 2460 to 2536 eV at 0.2-eV steps across the absorption edge. Spectra were evaluated and processed using the software packages SixPack (*49*) and Athena (*50*). Spectra from Ryugu and meteorites were normalized to the post-edge by assigning a pre-edge baseline and fitting a spline through the post-edge, and an *E*_0_ value of 2470 eV was assigned. Collected spectra were compared to reference minerals using Linear Combination Fitting (LCF) from 2467 to 2488 eV. A suite of >20 standards from our in-house library and the European Synchrotron Radiation Facility database were narrowed to elemental sulfur (S^0^), cysteine (C_3_H_7_NO_2_S), pyrrhotite [Fe_(1−*x*)_S], cysteic acid [HO_3_SCH_2_CH(NH_2_)CO_2_H] and gypsum (CaSO_4_·2H_2_O) used for LCF. The distinction of sulfur isomers, e.g., sulfide (S^2−^) and sulfate (S^6+^) (pyrrhotite versus troilite and gypsum versus bassanite/anhydrite, respectively) was not part of this study. The LCF included only non-negative contributions and were normalized to unity from mineral references. The reference fits were allowed small energy shifts (<0.5 eV) to account for differences in collection at different beam lines. Spectral fit accuracy was accessed on the basis of completeness and goodness of fit, i.e., close to 100% (±5%) of the features were accounted for in LCF and minimized χ^2^ error analysis (table S1). Spectra fit with ≥5% elemental sulfur or thiol (C_3_H_7_NO_2_S) were closely scrutinized for asymmetrical or double peaks between 2472 and 2474 eV to ensure that spectra were not overfit and fit components were above spectral noise. Component fits of <0.1% were not included as this is below the limit of detection for XANES LCF of sulfur species. For GRA 95229 and Murchison, 5 spectra (of 143) could not be fit to near unity and therefore were excluded. For Ryugu XANES in A0070 on the sulfate grain, we show strong self-absorption effect that limits quantification of the S species, and the particle XANES spots are conservatively assigned >90% sulfate.

### Electron probe microanalysis methodology

The Ryugu particle A0070 pressed in indium was studied with the electron probe microanalysis (EPMA) to relocate the Ca-thiosulfate grain A0070_TSul#1 analyzed with synchrotron XRF (Fig. 2). We acquired x-ray intensity maps for the Kα lines of C, O, Cl, Na, K, Mg, Ca, Al, Ti. Si, Fe, P, Mn, S, Cr, Mn, Ni, Cu, Zn, and the Lα line of In [elemental x-ray lines for O, Na, Ca, Fe, and S were measured with the wavelength-dispersive spectrometry (WDS) detector] to characterize the general mineralogy of the particle and to identify additional S-bearing and Fe-poor domains. We used a 15-keV beam with 20-nA current and acquired 740 × 1200 pixels with a 5-μm probe diameter and a dwell time of 30 ms per pixel (total analysis time of 8 hours) to map the whole Ryugu particle A0070. The Ca-thiosulfate grain A0070_TSul#1 was subsequently mapped using 15-keV accelerating voltage, 15-nA probe current, and a dwell time of 20 ms per pixel with a focused probe resulting in an apparent pixel width of 24 nm.

### NanoSIMS 50L isotopic analysis: data collection and data processing

The use of the NanoSIMS 50L to measure the isotopes compositions of the Ca-thiosulfate grain and its coating is a unique set of coordinated measurements. They are stated below in the order in which they were conducted.

The NanoSIMS 50L at Caltech Microanalysis Center was used for the isotopic measurements. We initially tuned the instrument to map ^12,13^C, ^12^C^14^N,^12^C^15^N, and ^32,33,34^S isotopes centered on the thiosulfate grain located in A0070 to measure the C and N isotopic ratios of the grain coating. The standards that were used for tuning were gypsum and an organic-rich isotope 1-hydroxy standard [1-hydroxybenzotriazole hydrate (C_6_H_5_N_3_O.xH_2_O): δ^13^C = −29.9‰; δ^15^N = −6.12‰; (*51*)]. The grain was presputtered followed by a ~1-hour long map with a ~1-pA Cs^+^ current to acquire an isotopic map. We used the normal-incidence electron gun for charge compensation for all analyses. The instrument apertures/slits setup is D1#3 and ES#2 with no AS or EnS. The maps were acquired at 256 × 256 pixels, 15 frames, and 600-μs dwell time per pixel. The mass resolving powers on the electronmultipliers were high enough to resolve the masses of interest from their adjacent interferences (e.g., ^12^C^15^N from ^13^C^14^N).

The sulfur ^32,33,34^S isotopes were measured with the NanoSIMS 50L in the isotope mode with a ~1.5-pA Cs^+^ current, using established procedures (*52*). Owing to very high-count rates (and ion yield) of the Canyon Diablo troilite and the fact that we were measuring a sulfate grain, we used two glass isotopic standards with low concentrations of S to quantify the S isotopic composition of the sulfate grain. The synthetic glasses used for tuning and calibration were EGT17-01 [2292–parts per million (ppm) sulfur with EPMA] and TNR14-1 (3387-ppm sulfur with EPMA) (*53*). The instrument apertures/slits setup is D1#3 and ES#2 with no AS or EnS. The beam raster size is 3 μm by 3 μm, with 64 × 64 pixels, and 268-μs dwell time per pixel. We measured 3 gypsum runs (20 min each), with standard deviations of 3.0‰ (1σ) for δ^33^S and 1.5‰ (1σ) for δ^34^S. For the isotopic map of the Ryugu sulfate grain, we got about 0.8‰ (1σ) for δ^34^S across the whole area of 8 μm by 8 μm, and about 2‰ (1σ) for δ^33^S, with about 1 hour’s collection time. The stable isotope of ^36^S was not measured because it occurs at 0.01%, and its measurement at the same precision as δ^33^S would take ~8 times longer.

The oxygen isotope data on the Ryugu thiosulfate A0070_TSul#1 were generated on the NanoSIMS 50L at Caltech using techniques that have been previously described (*54*). The oxygen isotopes ^16,17,18^O were measured in isotope mode with a Cs^+^ beam of ~3-pA current, a mass resolving power of over 9000 for mass ^17^O using D1#2, ES#4, AS#2 and EnS#1, to fully resolve it from the much higher ^16^O^1^H interference peak. The beam raster size is 3 μm by 3 μm, with 64 × 64 pixels, and 254-μs dwell time per pixel. For tuning and calibration, San Carlos (SCOL) and Eagle Station olivine standards with known and reported oxygen isotopic compositions (*55*, *56*) were used, although those reported in Fig. 2A are corrected by SCOL.

### Geochemical modeling for evaluating formation and destruction of N-bearing thiols

Geochemical modeling with input data, including activities of major cations, anions, and mineral species, assumed from (*3*) and constrained by known CC meteorites was used to calculate thermodynamic stability fields and predominance of sulfur species using the Act2 program in Geochemist’s Workbench V10.0.10 (*57*). Activities of species as yet unreported were assigned a log activity of 10^−3^, an assumption within the known constraints of C-type asteroids. Thermodynamic data were obtained from the internally consistent, built-in database (thermo.dat.v8.R7) included in the Act2 program (*58*, *59*). Activity coefficients were calculated in the dilute (<1 M) aqueous system according to the “B-dot” equation, an extended form of the Debye-Hückel equation (*60*). Model conditions were inclusive of temperatures from 0° to 150°C, pressures from 5 to 100 mbar (pressures in small asteroids should be <30 mbar), included aqueous species Ca^2+^, Fe^2+^, HCO_3_^+^, HPO_4_^2−^, NH_3_(*aq*), Mg^2+^, and K-feldspar as the dominant silicate solid phase. Precipitation of thermodynamically stable pyrite was suppressed in the model as it is not expected or observed in the mineralogical characterization of Ryugu (*3*) or other C-type asteroids, and to allow the examination of the predicted (meta)stability of aqueous sulfur phases. Suppression of mineral pyrite in the model results in the meta-stable fields of H_2_S(*aq*) and S(*s*) being visualized. Despite the (valid) assumptions of solute activities and the equations treating the activity coefficients, the broad predominance field of stable methionine across the wide pH range at all low-O_2_ conditions demonstrates thiol species are thermodynamically permissible.

## Supplementary Materials

### This PDF file includes:

Materials and Methods Figs. S1 to S10 Tables S1 and S2 References
