# Supplementary material for: Evidence of both molecular cloud and fluid chemistry in Ryugu regolith
Source: Sci Adv. 2024 Jul 24;10(30):eadp3037. doi: 10.1126/sciadv.adp3037 (PMC11268420; doi:10.1126/sciadv.adp3037)
Supplement: Supplementary file 1 — Materials and Methods Figs. S1 to S10 Tables S1 and S2 References [file sciadv.adp3037_sm.pdf]

Science Advances
AAAS

Supplementary Materials for

Evidence of both molecular cloud and fluid chemistry in Ryugu regolith

Maitrayee Bose *et al.*

Corresponding author: Maitrayee Bose, Maitrayee.Bose@asu.edu

Sci. Adv. **10**, eadp3037 (2024)
DOI: 10.1126/sciadv.adp3037

This PDF file includes:

Materials and Methods
Figs. S1 to S10
Tables S1 and S2
References

A. Ryugu Sample Handling and Preparation for Spectroscopic and Imaging analysis.

Prior to handling, mounting, and measurements of the Ryugu particles, we tested all the materials to be used for the mounting of the Ryugu particles for their S abundance, including the cyanoacrylate adhesive (Gorilla Super Glue Gel) and polyethylene plastic film (Kapton) thin film (3 μm) across several mm with a 5×5 μm^2 resolution, which each showed trace to no S in their respective micro X-ray absorption near edge structure (XANES) spectra (Figure S1).

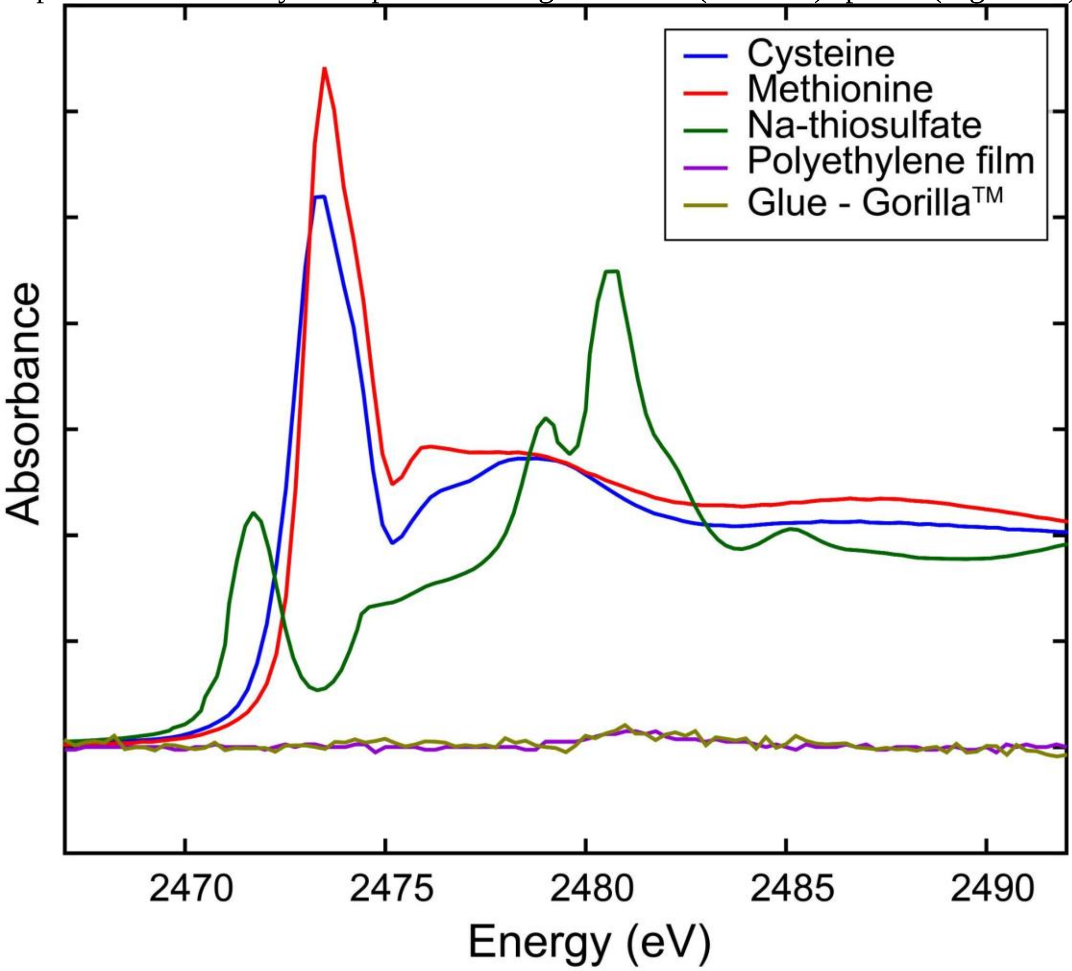

Fig. S1. *A comparison of the XANES spectra acquired on the polyethylene film, Gorilla glue(™) and reference standards sodium thiosulfate, cysteine, and methionine. Sodium thiosulfate was used as the in-line calibrant, with the first peak (S-S) assigned to 2472.04 eV.*

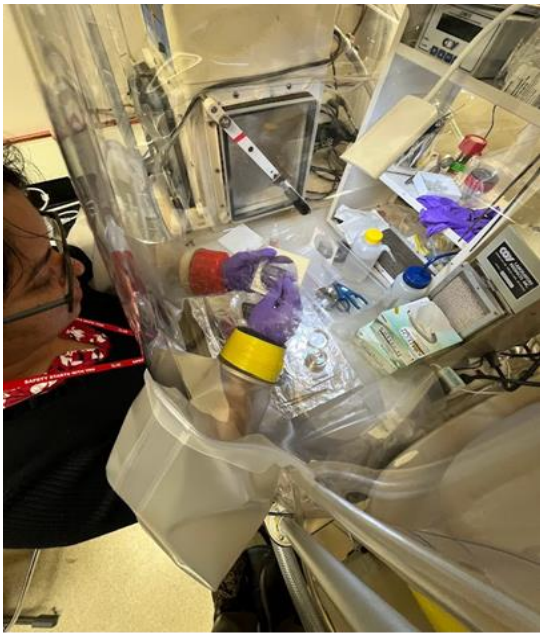

Fig. S2. *Opening JAXA container in anaerobic chamber.*

Ryugu particles were mounted in 3 different configurations in the anaerobic chamber (Figure S2):
1. Ryugu particle A0070 was affixed onto the thin film and the film was firmly adhered to a metal ring, which was mounted into the holder at the SLAC beamline, SSRL 14-3. During this process, the primary fragment of A0070 broke into 3 pieces and produced a fine powder. All 3 pieces (named A, B, C) were subsequently placed on the film without any loss.
2. The fine <1 mm-sized particles of A0070 that came loose during the mounting were collected and placed on sulfur-free mylar adhesive tape and exposed to air for ~2 hours to probe the effect of O_2 exposure on the sulfur speciation.
3. An indium mount was prepared and Ryugu particle A0093 was pressed into the indium and subsequently polished with diamond cloths (grit size ~9µm) within the anaerobic chamber. The main mass of the particle in the mount was divided into 4 quadrants and quadrant Q1 and Q2 were mapped. Additionally, a small satellite S1 mass was also mapped.

The primary particles of A0070 and A0093 were kept in the anaerobic chamber while standards were measured (e.g., Figure S1), and then moved in an N_2-filled container to the He-purged sample chamber at the beamline. Therefore, samples prepared by procedures 1 and 3 were not exposed to O_2 prior to or during XANES and synchrotron micro-X-ray fluorescence (µXRF) measurements.

The XANES spectra acquired on the Ryugu particle A0070 exposed to air (sample preparation step 2) were compared to those not exposed to air (steps 1 and 3), to confirm negligible speciation alteration (oxidation) due to short duration atmospheric exposure. Although the remaining data presented here are from samples not exposed to O_2, these confirmation data allow us to understand how sulfides can potentially transform under laboratory or storage settings. Figure S3 shows that S XANES spectra in particles exposed to air show a contribution at the sulfate peak energy with different intensities. The particles with intentional O_2 isolation, where care was taken to eschew air exposure, show similar sulfide and sulfate features, including those that do not contain a sulfate peak. This is interpreted as co-location of sulfide and sulfate grains at the spatial scale of the X-ray beam (~5 μm^2), and redoximorphic alteration of the particles. More importantly, the relative proportions of sulfur species typically found in terrestrial bio-residues (e.g., *62*) is very different from the distributions of sulfur species in our results; this is convincing evidence that our sample observations are unaffected by terrestrial contamination.

After XANES and XRF measurements at SLAC were complete, the film with the particles were stored (freshly N_2 purged) in the original JAXA container and returned to Arizona State University (ASU) and stored in an anaerobic chamber. The indium mount containing particle

A0093 was stored in an N_2 purged desiccator. Prior to elemental mapping with the electron microprobe, particle A0070 was remounted by pressing-in indium. The film was peeled from the sample surface, after pressing in indium. Finally, once the elemental maps and backscattered electron images had been acquired, and the sulfate grain (Figure 1a) had been located in A0070, the sample was sent to the Nano-scale Secondary Ion Mass Spectrometry (NanoSIMS) laboratory at the California Institute of Technology for S and O isotopic analysis. The mount containing the Ryugu particle A0070 was immediately placed in the airlock of the NanoSIMS instrument (with vacuum better than 5×10^{-7} torr).

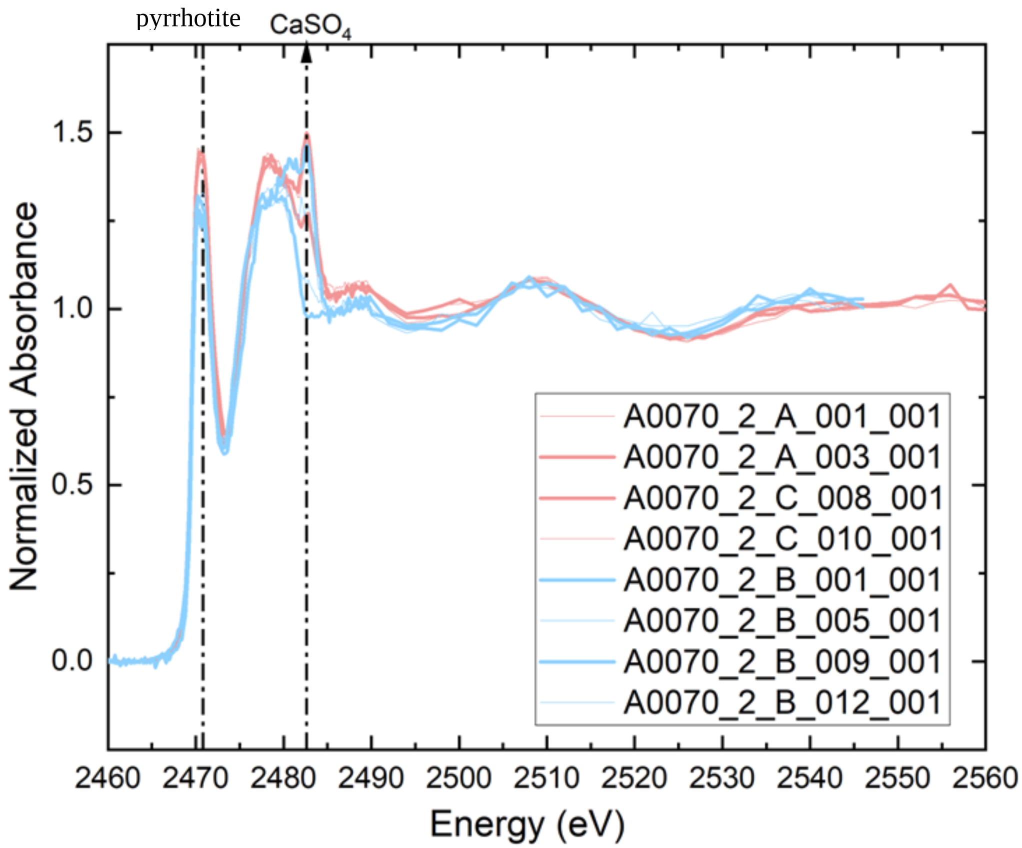

Fig. S3. *Comparison of the spectra from A0070 particles exposed to air (pink) and those not exposed to air (blue). The location of the pyrrohotite and Ca-sulfate peaks are shown by lines. Sample labels A, B, and C indicate preparation configuration 1, 2, and 3, respectively.*

For comparative analyses, XANES and XRF data were also acquired on thin sections of Murchison and Graves Nunataks (GRA) 95229 meteorites on loan from NASA Johnson Space Center. These were specifically chosen because of the less-severe terrestrial alteration that most CI carbonaceous chondrites (CCs) have experienced.

B. Micro-XRF and XANES.

B1. Methodology and Data Collection.

Five areas in Murchison and eight areas in GRA 95229 thin sections, each ≤ 1 × 1 mm^2 was investigated using μXRF and μXANES (Figure 4). The areas probed were inter-chondrule matrix and fine-grained rims around calcium, aluminum-rich inclusions and chondrules. Smaller areas were measured at multiple energies for spatial analysis of S species with stacked maps collected across the sulfur absorption edge. Spectra collected in each mapped area were designated with markers. Markers in Figures S5a and S5b use color coding to denote the presence or absence of sulfide, sulfate, and thiol based on linear combination fitting (LCF) to reference mineral spectra.

GRA 95229 and Murchison spectra with prominent elemental S^0 or HS^- spectral features were used in Figure S6 to show the potential higher energy thiol shoulder on the S^0 peak. In the cases where LCF resulted in component weights of < 1% with large relative errors, fits were repeated with the low contribution component omitted to investigate the necessary contribution of that component to the fit by evaluation of the fit sum and χ^2, the goodness of fit change. The best fit was assigned to the LCF with the lowest χ^2 and fewest components and was reported. Scrutinizing the LCF, with multiple iterations, was necessary for quantitative analysis and to ensure the consistency of results.

B2. Results from Murchison and GRA 95229. Areas mapped with μ-XRF from Murchison and GRA 95229 are shown with yellow bounding boxes over the reflected light photomicrograph (Figures S4a and S4b). Thick lines indicate coarse maps while thin lines are used to show multiple energy fine-scale map areas.

We observe co-localization of multiple sulfur species in Murchison and GRA 95229 (Figures S5a and S5b). GRA 95229 exhibits peaks of mono- and di-sulfides, sulfate, and a broad peak around 2472.5 eV corresponding to elemental sulfur and/or thiol. This peak at 2472.5 eV has been previously reported in Murchison (*20, 42*) but is much more prominent and abundant in GRA 95229 (Figure S6). The elemental sulfur and thiol peaks at about 2472.5 eV were shown to consistently co-exist with sulfate species within the 3 μm^2 pixel in the region mapped in the best resolution available at the time (Figures S5, S6), and generally exist in areas with relatively lower silicon content. We also identified a large ~120×260 μm^2 silicate grain, rich in aluminum in the matrix of GRA 95229, which contains monosulfide (troilite) and sulfate. Phosphides and calcium sulfates could be possible phases for the large assemblage found in GRA 95229. Smaller domains (< 15 μm in size) rich in phosphorus and calcium are distributed throughout this large grain. The abundance of troilite is higher in Murchison than in GRA 95229. In Murchison, the elemental sulfur and thiol peak at 2472.5 eV is always present with monosulfdes (e.g., mackinawite [$(Fe,Ni)_{1+x}S$] or troilite [FeS]) and sulfate. Among the XANES spots collected, 37 % of spectra in the Murchison matrix and 20 % in the fine-grained rims around igneous inclusions show presence of the elemental sulfur and thiol peak at 2472.5 eV. In fact, the elemental sulfur and thiol peak intensity is clearly observed in a fine-grained rim in Murchison (Figure S7). The Fe, Ni sulfides are primarily within the inclusion, while the pure FeS present dominates the fine-grained rim and matrix. Finally, P-bearing assemblages were not identified in the regions probed in Murchison.

Qualitatively, thiol compounds appear to be more abundant in GRA 95229 than Murchison because of the presence of a broader peak, observed as a higher energy shoulder on the S^0 peak, at the relevant energies (2472–2474 eV). Aqueous alteration of GRA 95229 is limited to its

matrix. Therefore, the formation and subsequent destruction of thiols in meteorites during the secondary alteration process is a possibility. When thiol is present, it is always with sulfate at the micron scale, and if the sulfate is asteroidal in origin, then by proxy, the thiol molecules could be produced by the same process. See the Main Text for a discussion on the formation pathways of thiols and sulfate. Thiols readily reduce to disulfides under specific reducing conditions, and this might explain the low abundance in Murchison.

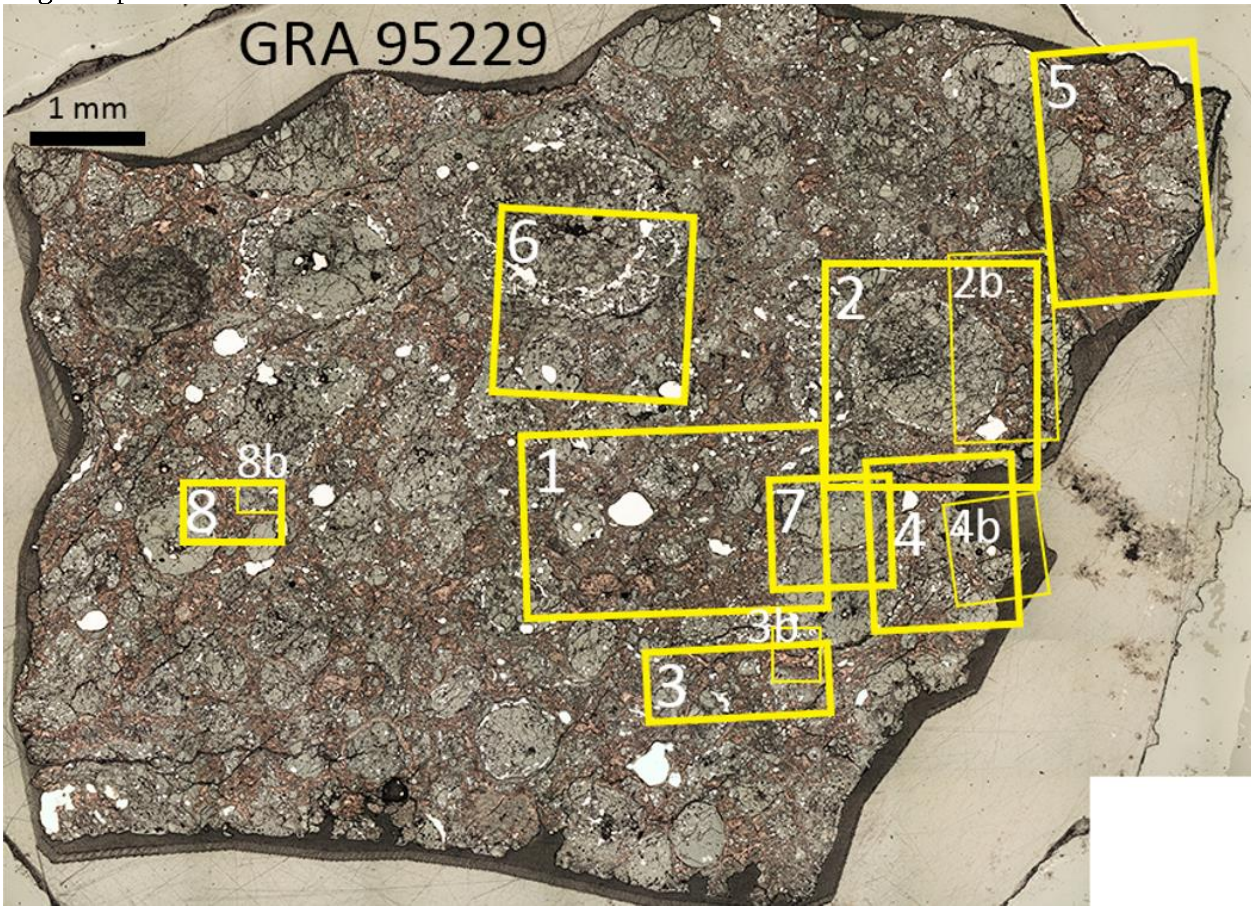

Fig. S4a. *Reflected light image of the GRA 95229 thin section. Areas measured for sulfur XANES and XRF are shown in yellow squares. Sub-areas are shown by thinner yellow squares. The resolution of the coarse maps are 10 µm² to 20 µm² per pixel with a dwell time of 10-20 ms. The resolution of the fine maps are 3 µm² per pixel with a dwell time of 50-75 ms. The elemental sulfur and thiol peaks occur in areas 1, 4, 5, 8.*

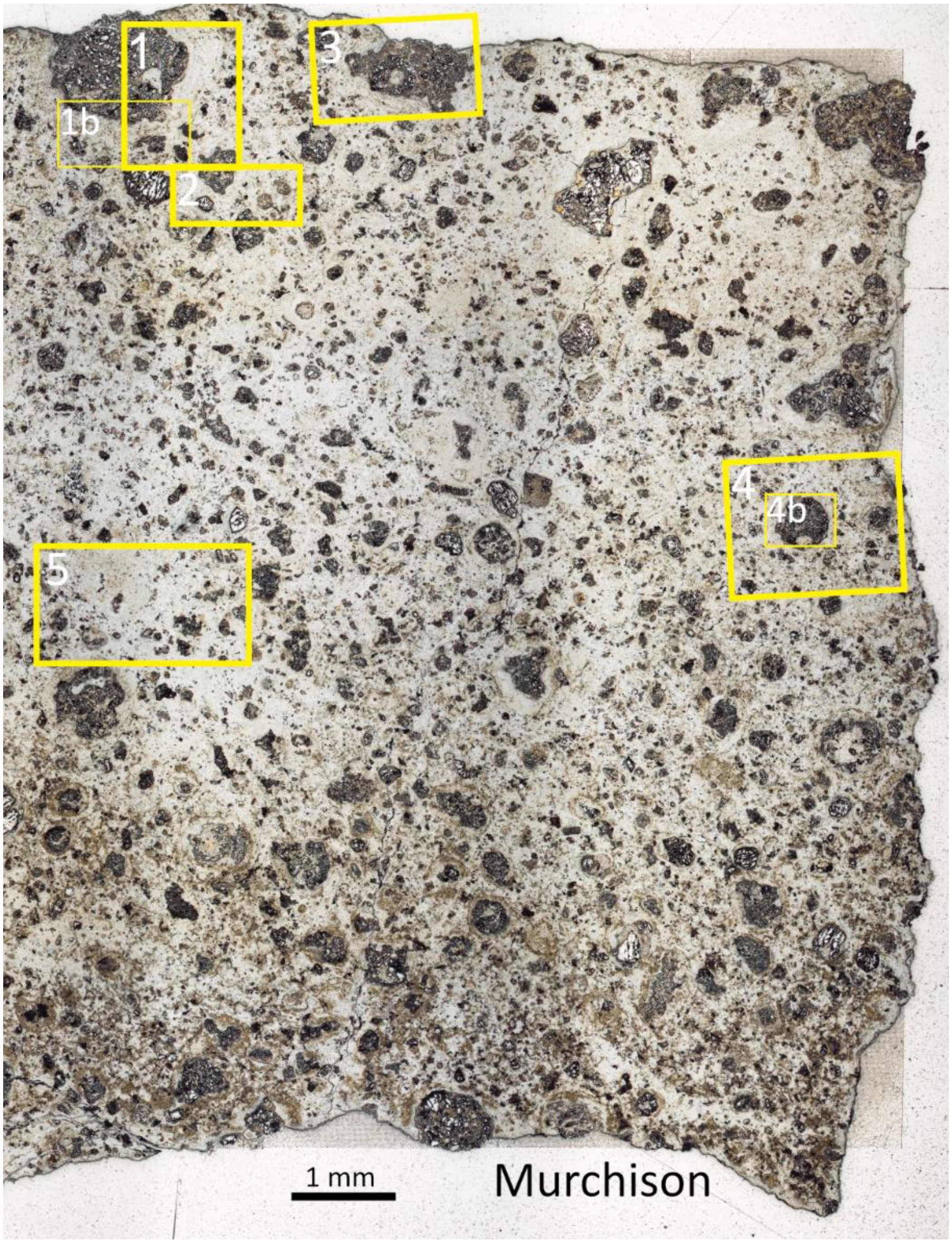

Fig. S4b. *Reflected light image of thin-section of Murchison. Areas measured for sulfur XANES and XRF are shown in yellow squares. The areas shown with yellow squares have step sizes of 3 µm and dwell times of 75 ms for each pixel. The elemental sulfur and thiol peaks occur in areas 3 & 4.*

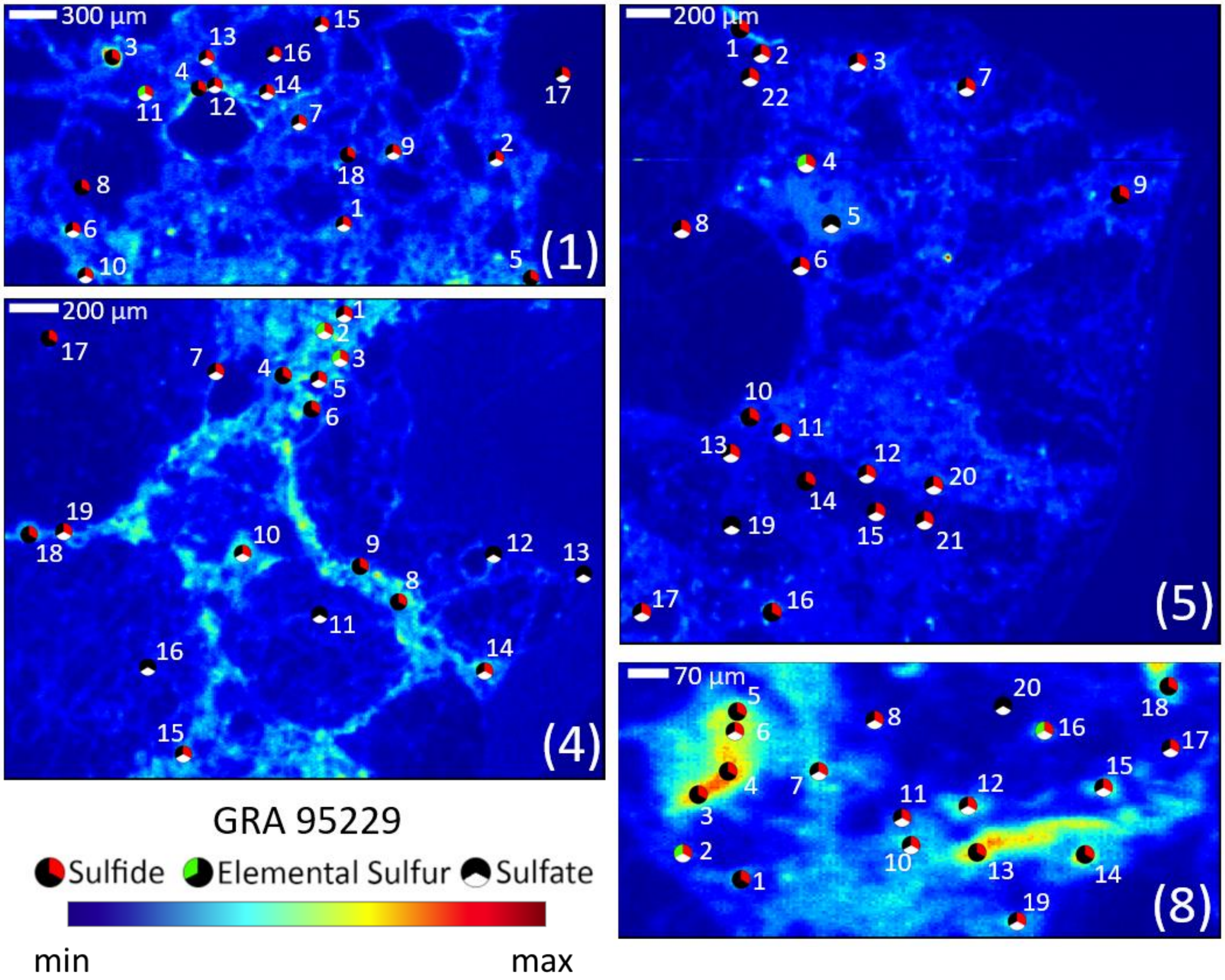

Fig. S5a. *Micro X-ray fluorescence (µ-XRF) heat maps for total sulfur (2500 eV) in GRA 95229. XANES spectra at points of interest show the distribution of sulfides, sulfates and elemental sulfur. Area number (bottom right) in each map corresponds to areas in Figure S4a. Markers denote location of XANES spectra. Points containing only, and combinations of, sulfide, elemental sulfur and sulfate were identified. The isopleth heat map color variation between maps is not equivalent.*

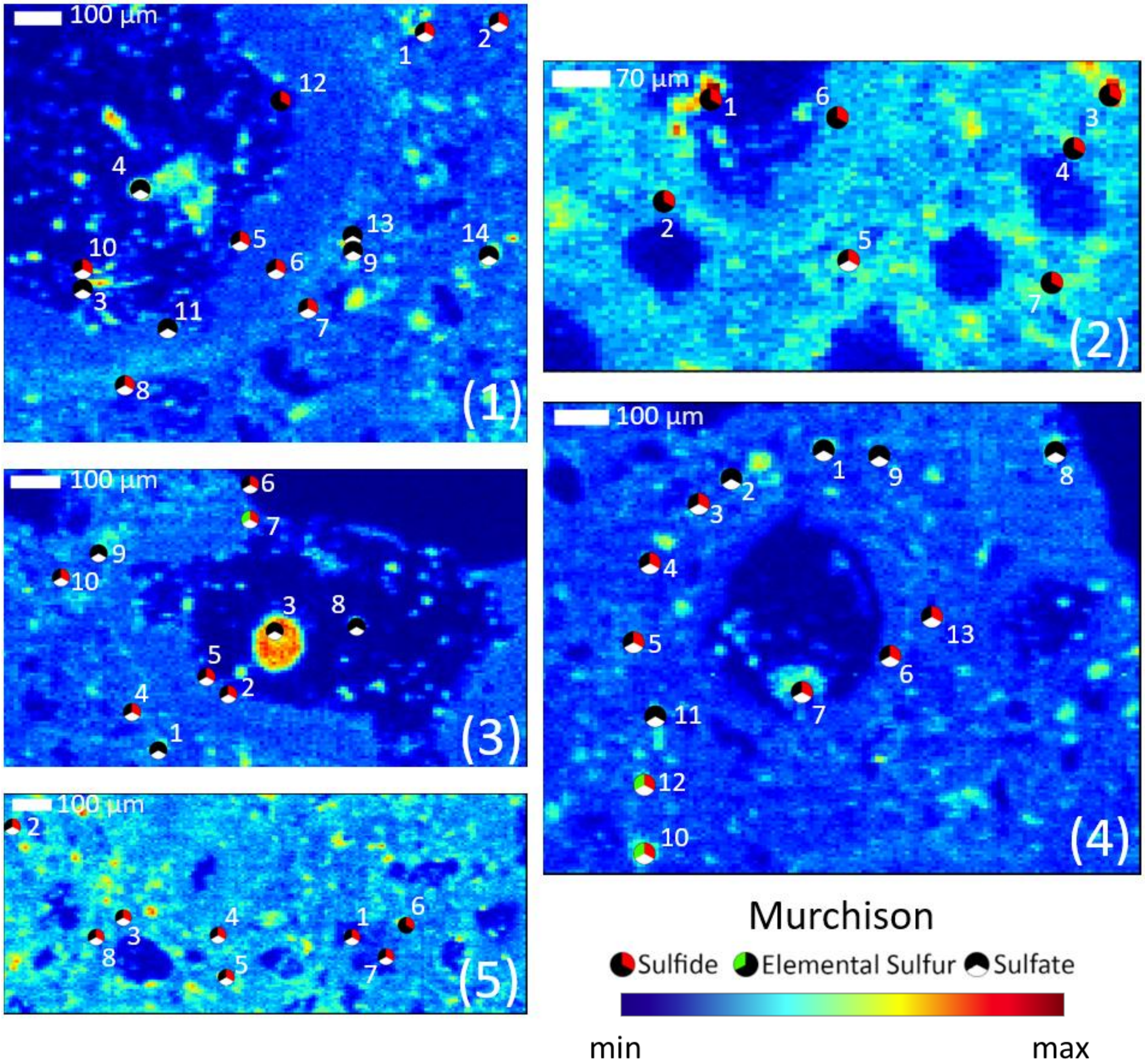

Fig. S5b. *X-ray Fluorescence (XRF) total sulfur heat maps (2500 eV) in Murchison. Each map number (bottom right) corresponds to Figure S4b. Markers denote XANES spot analysis, see S5a for a full description.*

Table S1: Linear combination fits of sulfur XANES in Ryugu particles and meteorites, Murchison and GRA 95229

Spot	Pyrrhotite	Elemental Sulfur	Thiol	Sulfate
**GRA 95229 Area 1**				
1	0.568 ± 0.016	0.080 ± 0.013	--	0.352 ± 0.005
2	0.721 ± 0.011	0.014 ± 0.010	0.035 ± 0.013	0.230 ± 0.004
3	0.914 ± 0.003	--	--	0.086 ± 0.003
4	0.947 ± 0.008	--	--	0.053 ± 0.002
5	0.947 ± 0.011	--	--	0.053 ± 0.003
6	0.317 ± 0.019	--	--	0.683 ± 0.005
7	0.555 ± 0.006	--	--	0.445 ± 0.015
8	0.987 ± 0.011	--	0.012 ± 0.014	--
9	0.617 ± 0.012	0.095 ± 0.010	--	0.288 ± 0.004
10	0.737 ± 0.012	0.095 ± 0.010	--	0.168 ± 0.003
**11**	**0.518 ± 0.015**	**0.077 ± 0.012**	**0.053 ± 0.017**	**0.352 ± 0.005**
12	0.522 ± 0.013	0.105 ± 0.011	0.017 ± 0.015	0.355 ± 0.004
13	0.620 ± 0.014	0.050 ± 0.011	0.031 ± 0.016	0.299 ± 0.004
14	0.831 ± 0.009	--	0.012 ± 0.011	0.158 ± 0.003
15	0.762 ± 0.010	0.030 ± 0.008	0.011 ± 0.010	0.196 ± 0.003
16	0.553 ± 0.023	0.086 ± 0.011	--	0.361 ± 0.005
17	0.726 ± 0.011	0.026 ± 0.009	0.027 ± 0.012	0.222 ± 0.003
18	0.912 ± 0.006	--	--	0.088 ± 0.002
26	0.515 ± 0.014	0.054 ± 0.012	0.031 ± 0.016	0.398 ± 0.005
27	0.516 ± 0.051	0.098 ± 0.010	0.032 ± 0.018	0.353 ± 0.004
**28**	**0.600 ± 0.016**	**0.028 ± 0.013**	**0.046 ± 0.018**	**0.326 ± 0.005**
29	0.782 ± 0.010	--	0.012 ± 0.011	0.207 ± 0.003
**GRA 95229 Area 4**				
1	0.568 ± 0.017	0.097 ± 0.014	--	0.335 ± 0.005
**2**	**0.593 ± 0.017**	**0.058 ± 0.015**	**0.026 ± 0.020**	**0.322 ± 0.006**
3	0.846 ± 0.011	--	0.070 ± 0.013	0.084 ± 0.003
4	0.904 ± 0.011	--	0.044 ± 0.013	0.052 ± 0.003
5	0.899 ± 0.011	--	--	0.100 ± 0.004
6	0.806 ± 0.012	0.013 ± 0.011	--	0.181 ± 0.004
7	0.867 ± 0.010	0.028 ± 0.009	--	0.105 ± 0.003
8	0.932 ± 0.015	--	--	0.068 ± 0.005
9	0.925 ± 0.009	--	0.043 ± 0.011	0.031 ± 0.003
10	0.735 ± 0.015	0.029 ± 0.013	--	0.236 ± 0.005
11	0.355 ± 0.023	--	0.027 ± 0.027	0.619 ± 0.007
12	0.112 ± < DL	--	0.023 ± < DL	0.865 ± < DL
13	0.322 ± 0.069	--	0.124 ± 0.081	0.554 ± 0.022
14	0.880 ± 0.022	--	--	0.120 ± 0.004

15	0.691 ± 0.018	0.040 ± 0.016	--	0.268 ± 0.006
16	0.133 ± < DL	--	0.022 ± < DL	0.845 ± < DL
17	0.859 ± < DL	--	0.141 ± < DL	--
18	0.880 ± 0.017	--	--	0.120 ± 0.005
19	0.817 ± 0.020	--	--	0.183 ± 0.006
**GRA 95229 Area 5**				
1	0.797 ± 0.015	0.027 ± 0.013	--	0.176 ± 0.004
1	0.668 ± 0.020	0.031 ± 0.018	--	0.302 ± 0.007
2	0.841 ± 0.010	--	0.061 ± 0.012	0.098 ± 0.004
3	0.226 ± 0.026	0.217 ± 0.024	0.060 ± 0.031	0.497 ± 0.009
**4**	**0.712 ± 0.016**	**0.014 ± 0.014**	**0.043 ± 0.019**	**0.231 ± 0.005**
5	0.696 ± 0.013	0.083 ± 0.011	--	0.221 ± 0.004
6	0.687 ± 0.021	0.090 ± 0.010	--	0.223 ± 0.004
7	0.894 ± 0.012	--	--	0.106 ± 0.004
8	0.972 ± 0.011	--	--	0.028 ± 0.004
9	0.973 ± 0.012	--	--	0.027 ± 0.003
10	0.612 ± 0.022	0.117 ± 0.011	--	0.272 ± 0.006
11	0.802 ± 0.012	0.016 ± 0.010	--	0.182 ± 0.005
12	0.819 ± 0.022	0.024 ± 0.019	--	0.156 ± 0.006
13	0.614 ± 0.023	0.028 ± 0.020	--	0.358 ± 0.007
14	0.969 ± < DL	--	0.011 ± < DL	0.020 ± < DL
15	0.934 ± < DL	--	--	0.066 ± < DL
16	0.630 ± 0.024	--	0.180 ± 0.028	0.191 ± 0.007
17	0.569 ± 0.021	0.021 ± 0.018	0.023 ± 0.024	0.388 ± 0.006
18	0.864 ± < DL	--	--	0.136 ± < DL
19	0.713 ± 0.028	0.028 ± 0.025	--	0.259 ± 0.008
20	0.681 ± 0.037	0.043 ± 0.033	--	0.276 ± 0.011
21	0.801 ± < DL	--	--	0.199 ± < DL
22	0.610 ± 0.022	0.084 ± 0.018	--	0.306 ± 0.007
**GRA 95229 Area 8**				
1	0.862 ± 0.025	--	0.046 ± 0.029	0.092 ± 0.008
**2**	**0.778 ± 0.017**	**0.022 ± 0.015**	**0.035 ± 0.021**	**0.165 ± 0.005**
3	0.912 ± 0.012	--	0.054 ± 0.012	0.033 ± 0.023
4	0.921 ± 0.012	--	0.033 ± 0.014	0.046 ± 0.004
5	0.876 ± 0.013	--	0.042 ± 0.015	0.083 ± 0.004
6	0.886 ± 0.013	0.016 ± 0.012	0.019 ± 0.016	0.077 ± 0.004
7	0.760 ± 0.016	0.037 ± 0.014	0.020 ± 0.019	0.183 ± 0.005
8	0.432 ± 0.028	0.029 ± 0.024	0.016 ± 0.033	0.523 ± 0.008
9	0.823 ± 0.044	0.018 ± 0.009	0.053 ± 0.016	0.106 ± 0.004
10	0.709 ± 0.045	--	0.114 ± 0.023	0.177 ± 0.007
11	0.612 ± 0.022	0.021 ± 0.019	0.061 ± 0.026	0.307 ± 0.007
12	0.777 ± 0.008	--	--	0.223 ± 0.011

13	0.923 ± < DL	--	--	0.077 ± < DL
14	0.917 ± < DL	--	0.047 ± < DL	0.037 ± < DL
15	0.744 ± 0.019	0.044 ± 0.017	--	0.212 ± 0.041
**16**	**0.322 ± 0.037**	**0.082 ± 0.032**	**0.058 ± 0.043**	**0.537 ± 0.011**
17	0.753 ± 0.008	--	--	0.247 ± 0.008
18	0.893 ± 0.021	--	--	0.107 ± 0.007
19	0.759 ± 0.027	--	0.078 ± 0.027	0.163 ± 0.044
20	0.480 ± 0.029	0.033 ± 0.025	--	0.486 ± 0.009
**Murchison Area 1**				
1	0.609 ± 0.021	--	--	0.391 ± 0.029
2	0.447 ± 0.017	--	--	0.553 ± 0.017
3	0.932 ± 0.019	--	--	0.068 ± 0.006
4	0.969 ± < DL	--	0.031 ± < DL	--
5	0.649 ± 0.023	0.023 ± 0.020	--	0.330 ± 0.007
6	0.641 ± 0.007	--	--	0.359 ± 0.007
7	0.873 ± 0.018	--	--	0.126 ± 0.006
8	0.880 ± 0.019	--	--	0.120 ± 0.006
9	0.868 ± 0.001	--	0.016 ± 0.014	0.116 ± 0.004
10	0.666 ± 0.026	--	--	0.334 ± 0.037
11	0.980 ± 0.015	--	--	0.020 ± 0.005
12	0.476 ± 0.020	--	--	0.524 ± 0.020
13	0.892 ± 0.011	--	--	0.108 ± 0.004
14	0.967 ± < DL	--	0.013 ± < DL	0.020 ± < DL
**Murchison Area 2**				
1	0.991 ± < DL	--	--	0.009 ± < DL
2	0.931 ± 0.019	--	--	0.069 ± 0.006
3	0.983 ± < DL	--	--	0.017 ± < DL
4	0.949 ± < DL	--	0.050 ± < DL	--
5	0.483 ± 0.055	--	--	0.527 ± 0.005
6	0.839 ± 0.014	--	--	0.161 ± 0.008
7	0.850 ± 0.007	--	--	0.150 ± 0.007
**Murchison Area 3**				
1	0.959 ± 0.008	--	0.029 ± 0.010	0.012 ± 0.003
2	0.711 ± 0.016	0.022 ± 0.014	--	0.267 ± 0.005
3	0.975 ± 0.020	--	--	0.026 ± 0.006
4	0.590 ± 0.016	--	--	0.410 ± 0.005
5	0.427 ± 0.021	--	--	0.574 ± 0.006
6	0.705 ± 0.008	--	--	0.295 ± 0.008
**7**	**0.639 ± 0.021**	**0.050 ± 0.018**	**--**	**0.311 ± 0.007**
8	0.973 ± < DL	--	--	0.027 ± < DL
9	0.898 ± 0.016	--	0.031 ± 0.019	0.072 ± 0.005
10	0.659 ± 0.020	0.044 ± 0.018	--	0.296 ± 0.006

**Murchison Area 4**				
1	0.890 ± 0.015	--	0.034 ± 0.018	0.075 ± 0.005
2	0.996 ± 0.017	--	--	0.004 ± 0.005
3	0.747 ± 0.015	--	--	0.254 ± 0.005
4	0.925 ± < DL	--	--	0.075 ± < DL
5	0.505 ± 0.099	0.032 ± 0.008	--	0.463 ± 0.004
6	0.975 ± < DL	--	0.005 ± < DL	0.020 ± < DL
7	0.591 ± 0.017	--	--	0.409 ± 0.005
8	0.930 ± 0.017	--	--	0.070 ± 0.005
9	0.989 ± 0.013	--	--	0.011 ± 0.004
**10**	**0.808 ± 0.012**	**0.103 ± 0.010**	**--**	**0.089 ± 0.017**
11	0.974 ± < DL	--	0.019 ± < DL	0.009 ± < DL
**12**	**0.806 ± 0.024**	**0.049 ± 0.021**	**--**	**0.145 ± 0.034**
13	0.887 ± < DL	--	--	0.113 ± < DL
**Murchison Area 5**				
1	0.474 ± 0.034	--	--	0.526 ± 0.012
2	0.591 ± 0.011	--	--	0.409 ± 0.011
3	0.588 ± 0.035	--	--	0.412 ± 0.012
4	0.604 ± 0.018	--	--	0.396 ± 0.005
5	0.846 ± 0.016	--	--	0.154 ± 0.020
6	0.943 ± < DL	0.009 ± < DL	0.035 ± < DL	0.012 ± < DL
8	0.659 ± 0.014	--	0.020 ± 0.017	0.321 ± 0.005
**Ryugu A0070 Area A**				
1.1	0.966 ± 0.020	--	--	0.034 ± 0.004
1.2	0.948 ± 0.027	--	--	0.052 ± 0.005
2.1	0.960 ± 0.025	--	--	0.040 ± 0.005
2.2	0.956 ± 0.055	--	--	0.044 ± 0.011
3.1	0.967 ± 0.022	--	--	0.033 ± 0.004
4.1	0.983 ± 0.030	--	--	0.017 ± 0.006
4.2	0.957 ± 0.030	--	--	0.043 ± 0.006
5.1	0.957 ± 0.028	--	--	0.043 ± 0.006
5.2	0.964 ± 0.018	--	--	0.036 ± 0.004
6	0.929 ± 0.024	--	--	0.071 ± 0.005
7	0.944 ± 0.031	--	--	0.056 ± 0.006
8	0.915 ± 0.028	--	--	0.085 ± 0.008
9	0.938 ± 0.031	--	--	0.062 ± 0.006
10	0.933 ± 0.016	--	--	0.067 ± 0.005
**Ryugu A0070 Area B**				
1	1.000 ± 0.019	--	--	--
2 (2*)	0.979 ± 0.038	--	--	0.021 ± 0.007
3	0.939 ± 0.025	--	--	0.059 ± 0.005
4	0.947 ± 0.020	--	--	0.053 ± 0.006

5	0.998 ± 0.001	--	--	--
7.2	0.931 ± 0.027	--	--	0.068 ± 0.005
8	0.957 ± 0.021	--	--	0.043 ± 0.004
9	0.941 ± 0.035	--	--	0.059 ± 0.005
10	0.931 ± 0.035	--	--	0.065 ± 0.007
11.1	--	--	--	> 0.900
**11.1.1 (1*)**	**--**	**--**	**--**	**> 0.900**
**11.1.2**	**0.957 ± 0.021**	**--**	**--**	**0.043 ± 0.004**
**11.1.3 (3*)**	**--**	**--**	**--**	**> 0.900**
**11.1.4 (4*)**	**--**	**--**	**--**	**> 0.900**
**11.1.5 (5*)**	**--**	**--**	**--**	**> 0.900**
**11.1.6 (6*)**	**--**	**--**	**--**	**> 0.900**
12	0.964 ± 0.020	--	--	0.036 ± 0.004
13	0.957 ± 0.038	--	--	0.043 ± 0.008
**Ryugu A0070 Area C**				
8	0.953 ± 0.029	--	--	0.047 ± 0.006
9	0.947 ± 0.027	--	--	0.053 ± 0.007
10	0.947 ± 0.019	--	--	0.053 ± 0.005
11	0.927 ± 0.022	--	--	0.072 ± 0.004
**Ryugu A0093 Area Q1**				
1 (10*)	0.999 ± 0.016	--	--	--
2 (11*)	0.999 ± 0.015	--	--	--
3 (12*)	0.980 ± 0.013	--	--	0.020 ± 0.003
4	0.981 ± 0.020	--	--	0.019 ± 0.004
**5 (7*)**	**0.264 ± 0.037**	**--**	**0.678 ± 0.035**	**0.058 ± 0.010**
**Ryugu A0093 Area Q1sp5**				
**1.2 (8*)**	**--**	**--**	**1.000 ± < DL**	**--**
**2 (9*)**	**--**	**--**	**1.000 ± < DL**	**--**
3	0.991 ± 0.019	--	--	0.009 ± 0.003
**Ryugu A0093 Area Q2**				
9 (10*)	0.969 ± 0.027	--	--	0.030 ± 0.005
10 (11*)	0.948 ± 0.031	--	--	0.052 ± 0.006
11 (12*)	0.963 ± 0.022	--	--	0.037 ± 0.003
**Ryugu A0093 Area S1**				
6	0.842 ± 0.027	--	--	0.158 ± 0.005
7	0.998 ± 0.020	--	--	--
8	0.976 ± 0.021	--	--	0.024 ± 0.003

Spectra are normalized to 100 %. The area names correspond to Figures S4a, S4b, S5a, and S5b. The bold rows include datapoints that show a clear elemental sulfur and/or thiol peak in their XANES spectra. Abundances below the detection limits of 0.001 are shown by '--'. Where the 1σ uncertainty > component value, the component value is less constrained. Spectra in Fig. 1c are identified by "number(*)".

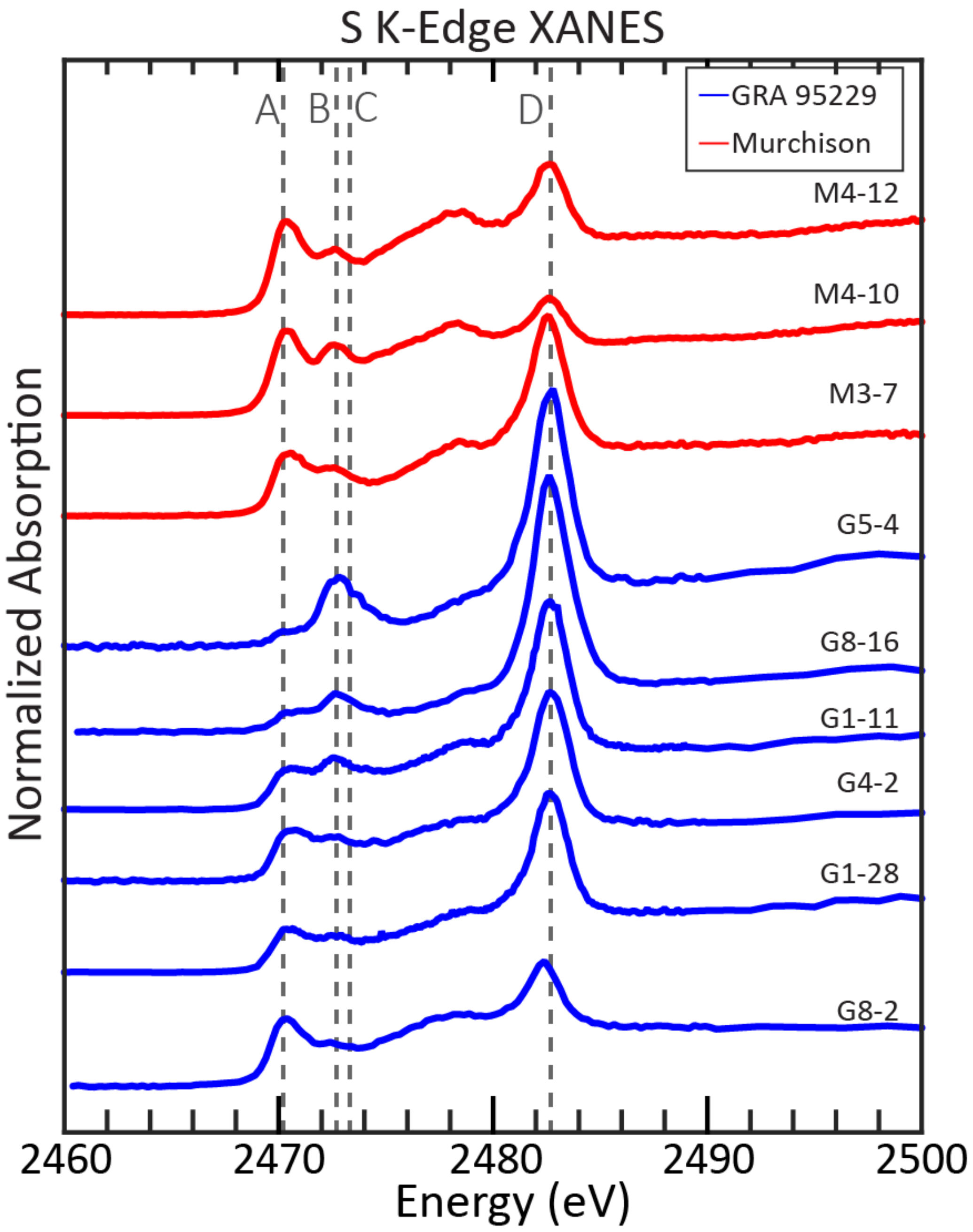

Fig. S6. *Murchison (red) and GRA 95229 (blue) XANES spectra with >5% elemental sulfur/thiol are shown. Dashed lines correspond to sulfide (A) at 2470.0 eV, elemental sulfur (B) at 2472.0 eV, thiol (C) at 2473.3 eV, and sulfate (D) at 2482.7 eV. We surmise that thiol is present in some of these domains, due to the asymmetry of the XANES spectral features at B and C. This asymmetry at B and C is more prominent in GRA 95229 than Murchison.*

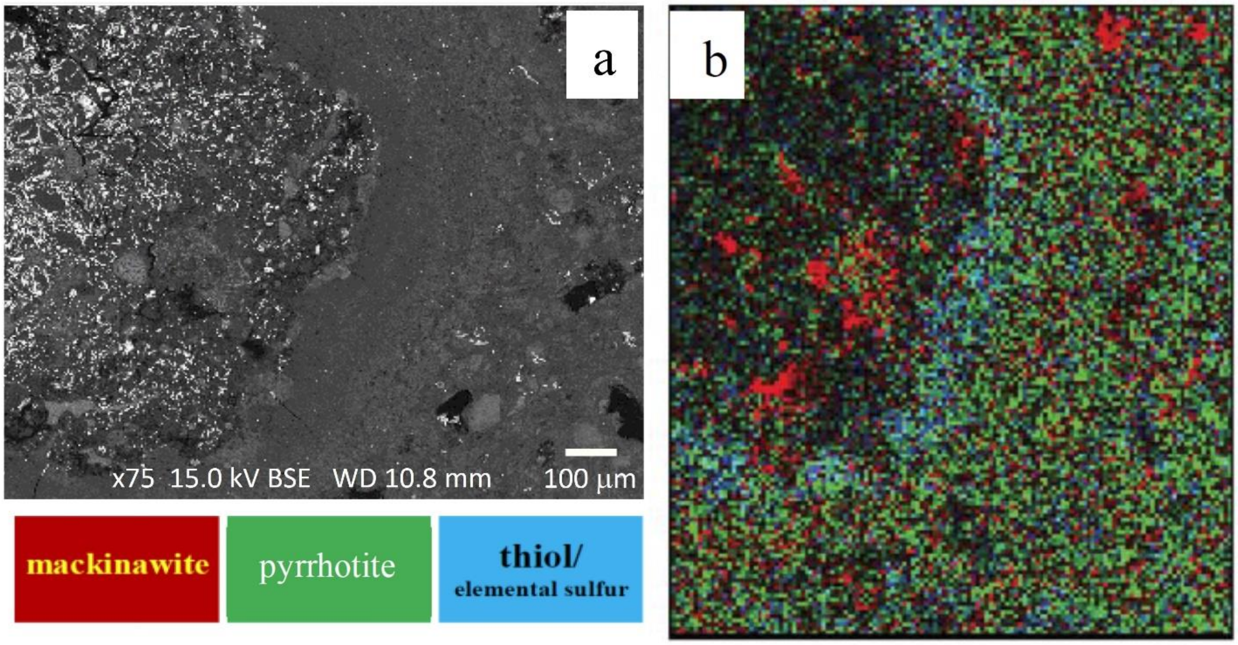

Fig. S7. *(a) Backscattered electron image of an area in Murchison showing an inclusion and its fine-grained rim. Scale bar is 100 µm. (b) Low-resolution (7×7 μm^2) µXRF sulfur oxidation state maps of the white rectangular area drawn in (a). The RGB map shows the presence of mackinawite in red, pyrrhotite in green and thiol/elemental sulfur in blue. The fine-grained rim is richer in thiol/ elemental sulfur than the matrix.*

B3. Results from Ryugu particles A0070 and A0093. Quantitative analysis of sulfur species by LCF of Ryugu XANES spectra is plotted in Figure S8, along with GRA 95229 and Murchison datasets. Components were constrained to be non-negative and the goodness of fit was evaluated by minimizing chi-squared ($\chi^2 = \sum(obs_i - exp_i)^2/exp_i$). Errors shown in Table S1 are ± 1σ. Of note are the thiol-rich domains (marked 7, 8, and 9; shown in Figure 1) that appear in Ryugu but not in GRA 95229 and Murchison.

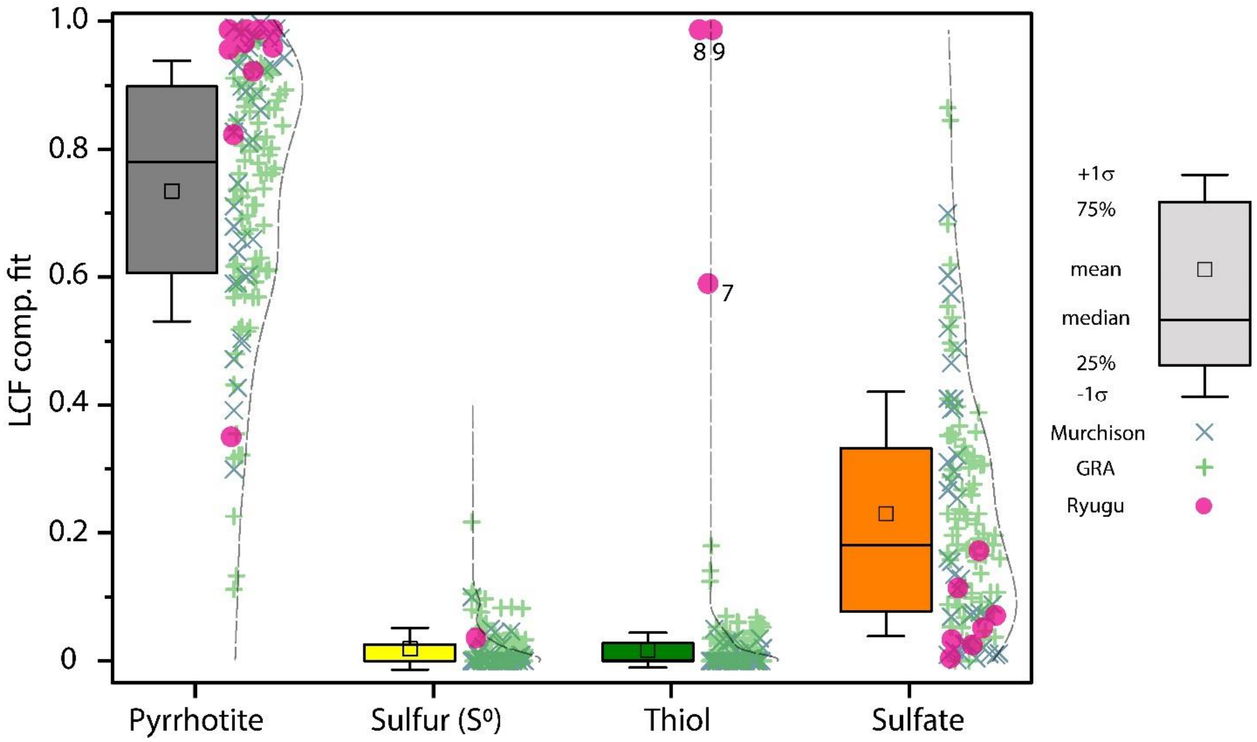

Fig. S8a. *Distribution of proportional linear combination fit (LCF) contributions from sulfur species identified in GRA 95229, Murchison and Ryugu particles based on XANES; data in Table S1. Each component contributed 0 – 1.0 to an individual S XANES spectra. Box and whisker plots show mean (square symbol), median (line in box), box boundaries are 25 % and 75 %, error bars show 1σ standard deviation of the data, individual fits are shown with 'x', '+' and 'o' markers, the dashed line bounding the data points shows the kernel smoothed fit.*

B4. Additional fits to A0093 spectra. Quantitative analysis of the S species in Ryugu particles was achieved with least-squares LCF of sulfur K-edge XANES. Minimizing the reduced chi-squared parameter by iterative variance of the components in the final fit results in a good description of the data. However, a good fit reveals that the data are a good match to the modelled reference spectra and does not indicate the appropriateness of the model compounds. LCF fits reference compounds and peak fitting assigns specific energy transitions. A limitation of LCF is that it is dependent on the reference database representing the samples being fit. Therefore, we have compared LCF to peak fitting of S XANES and determined that both approaches are appropriate and describe the Ryugu spectra well. Below, we show results for A0093_ln_sp5_007, A0093_ln_sp5_008, and A0093_ln_sp5_009 using peak fitting for completeness.

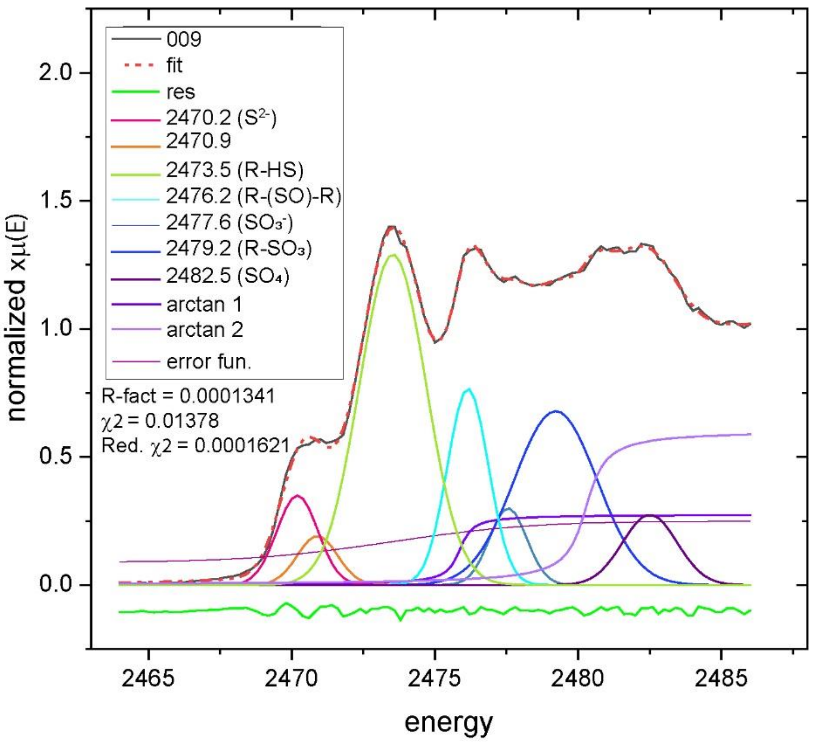

Figure S8b. Gaussian curve fitting for sulfur XANES at spot 7 from Figure 1B for Ryugu particle A0093. We employed additional Gaussian curve fitting method to spots 7–9 as a supplemental approach to LCF to assess the various S components. The confirmational analyses agreed with inorganic sulfide pyrrhotite, including the doublet peaks at 2470.2 and 2470.9 eV, as well as confirming the Ca sulfate by its main peak at 2482.5 eV. The main peak located at 2473.5 eV was a good fit to cysteine by LCF and confirmed to match the characteristic energy for the thiol (R-SH) functional group. The Gaussian curve fitting method suggested some additional details that were not accounted for in the LCF. Compounds identified by Gaussian curve fitting showed small contributions from methionine sulfoxide at 2476.2 eV (such as methionine sulfoxide), sulfone or sulfite at 2479.2 eV. These independently confirm the LCF results for the inorganic sulfide and sulfate spectra and organic thiol and thioester. Gaussian fit parameters: 10 lineshapes, 111 data points, 26 variables, R-factor = 0.0001654, reduced χ^2 = 0.0002449.

Energy (eV)	Height	Center (eV)	Sigma
2470.2	0.626(0.074)	2470.20(0.00)	0.715(0.033)
2470.9	0.334(0.086)	2470.90(0.00)	0.698(0.086)
2473.5	3.764(0.764)	2473.53(0.30)	1.163(0.040)
2476.2	1.378(0.434)	2476.15(0.72)	0.717(0.061)
2477.6	0.490(0.667)	2477.55(0.98)	0.653(0.269)
2479.2	2.455(0.958)	2479.21(1.96)	1.443(0.748)
2482.5	0.652(0.785)	2475.50(0.00)	0.947(0.242)
Arctan1	0.275(0.340)	2475.86(1.69)	1.443(0.748)
Arctan2	0.6040(0.197	2480.24(0.71)	0.500(0.000)
Err Func	0.078(0.214)	2473.5(15.04)	5.493(5.535)

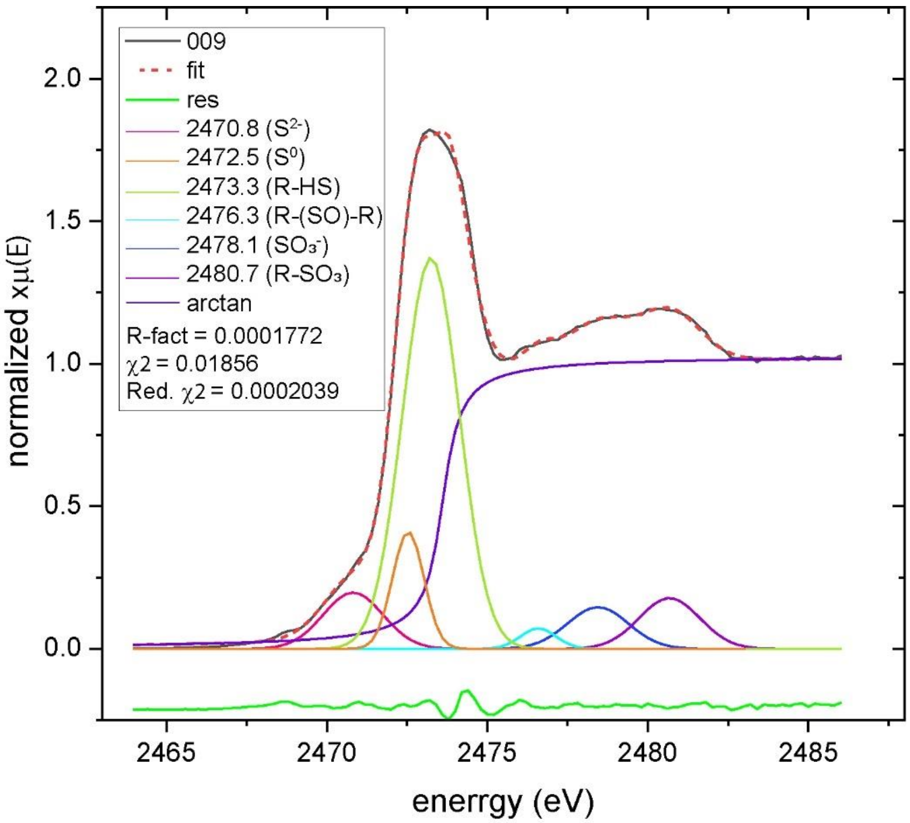

Figure S8c. Gaussian curve fitting for sulfur XANES at spot 8 from Figure 1B for Ryugu particle A0093. This spectrum was interpreted by LCF to be pure cysteine, the organic thiol species. Gaussian peak fitting shows that the main peak could be successfully fit by a combination of two peaks at 2472.5 eV and 2473.3 eV, well matched to cysteine (2473.5 eV) with a contribution from elemental sulfur (2472.6 eV), which was not found by LCF. Minor components also found by peak fitting included methionine sulfoxide (2476.6 eV) and sulfonate (2478.4 eV), and sulfone or sulfite (2480.7 eV). Fit parameters: 7 lineshapes, 111 data points and 20 variables, R-factor = 0.0001772, reduced χ^2 = 0.0002039.

Energy (eV)	Height	Center (eV)	Sigma
2470.8	0.460(0.630)	2470.82(33.75)	0.933(0.054)
2472.5	0.505 (0.144)	2472.53 (0.16)	0.490(0.049)
2473.3	3.029 (5.951)	2473.24(0.22)	0.881(0.079)
2476.6	0.099 (0.069)	2476.58(0.75)	0.552(0.147)
2478.4	0.347(0.108)	2478.44(0.77)	0.950(0.000)
2480.7	0.422(0.127)	2480.67(0.13)	0.949(0.082)
Arctan	1.029 (0.006)	2473.60(0.53)	0.414(0.044)

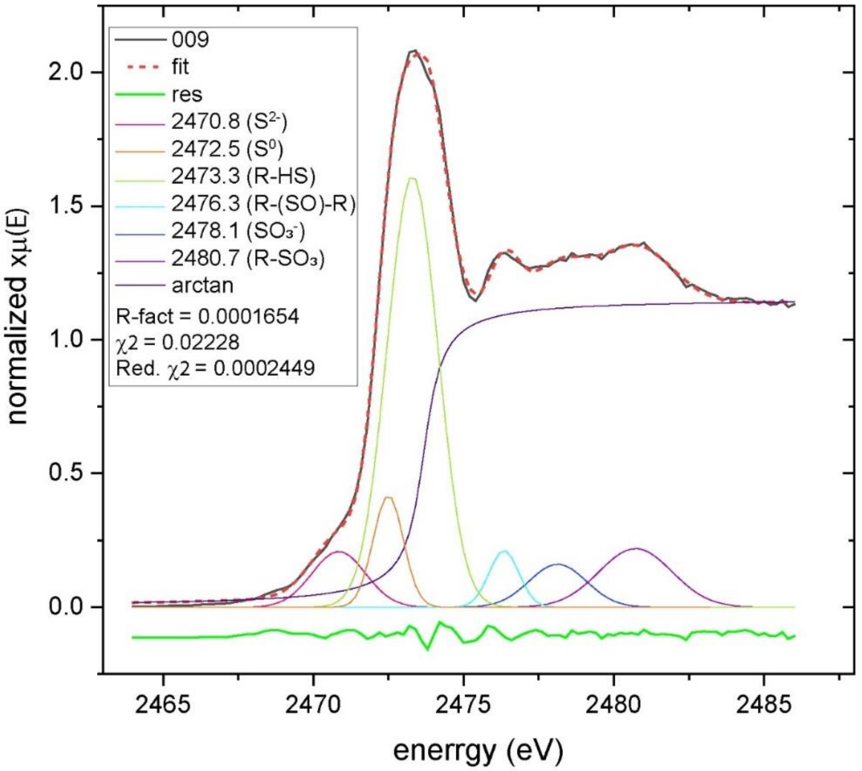

Figure S8d. Gaussian curve fitting for sulfur XANES at spot 9 from Figure 1B for Ryugu particle A0093. This spectrum was interpreted by LCF to be cysteine (thiol R-HS). Gaussian curve fitting indicated mostly cysteine (2473.3 eV), with minor contributions from monosulfide (2470.8 eV), elemental sulfur (2472.5 eV), sulfonate (2476.3 eV), sulfone (2478.1 eV), and sulfoxide (2480.7 eV). Sulfoxide R-(SO)-R, sulfone R-(SO_2)-R and sulfonate R-SO_3, and sulfate ester R-OSO_3 are typical oxidation products of organosulfides and thiols. The presence of these products with the sulfide/thiol indicate a partial oxidation process, and demonstrate that they shared a common origin. While the spatial and textural context supports the interpretation that this partial oxidation occurred on the parent body, and although extensive precautions were employed to eschew atmospheric contact, the possibility of partial oxidation post collection cannot be completely ruled out.

Energy (eV)	Height	Center (eV)	Sigma
2470.8	0.466(37.80)	2470.84(200.44)	0.893(0.052)
2472.5	0.525 (0.417)	2472.49 (0.31)	0.501(0.086)
2473.3	3.412(3.635)	2473.30(0.30)	0.843(0.520)
2476.3	0.264(0.123)	2476.34(0.20)	0.497(0.053)
2478.1	0.383(0.180)	2478.13(1.17)	0.950(0.000)
2480.7	0.666(0.167)	2480.72(0.17)	1.212(0.153)
Arctan	1.155(0.008)	2473.67(0.58)	0.452(0.049)

C. Results from EPMA EDS and X-ray intensity mapping.

C1. Methodology and Data Collection. Backscattered electron images and X-ray intensity maps of GRA 95229 were obtained at ASU using a JEOL JXA 8530F electron microprobe operated with an accelerating voltage of 15 keV and a probe current of 15 nA.
The X-ray intensity maps of the GRA 95229 and Murchison samples (of areas shown in Figures S4a and S4b) were conducted with the electron microprobe, after the acquisition of XANES and XRF data. We used an accelerating voltage of 15 keV, a probe current of 40 nA, a probe diameter of 2 µm, and a dwell time of 2 ms/pixel. The mapped X-ray intensities we obtained are for the Kα lines C, O, Cl, Na, K, **Mg**, **Ca**, Al, Ti. **Si**, **Fe**, P, Mn, **S**, Cr, Mn, Cu, and the Lα line of Au (elements in **bold** were measured with WDS)

C2. Results on GRA 95229 and Murchison. GRA 95229 is a 128.9 g CC Renazzo-type 2 (CR2) chondrite (*63*) that was recovered in 1995 by the Antarctic search for Meteorites (ANSMET) program (*64*). CR2 chondrites are distinguished by large, abundant porphyritic chondrules, several with igneous rims, a few refractory inclusions, abundant Fe metal, and fine-grained, hydrated matrix. GRA 95229 records minor terrestrial weathering (*65*) without evidence of elemental redistribution outside veinlets, minimal occlusion of primary porosity yet rust veinlets radiating from altered Fe-Ni metal grains occur, while no systematic differences in weathering were noted between interior and exterior Fe-Ni nodules (*66*). Common matrix minerals in GRA 95229 (Figure S9a) are amorphous Fe-Mg silicates, ferrolizardite, saponite, and FeNi sulfide, while forsteritic olivine, low-Ca pyroxene, Fe-oxide, and carbon-nanoglobules are accessory components according to (*66*). Notably, GRA 95229 contains fairly abundant presolar grains that occur concentrated in fine dust rims on chondrules (*67*).

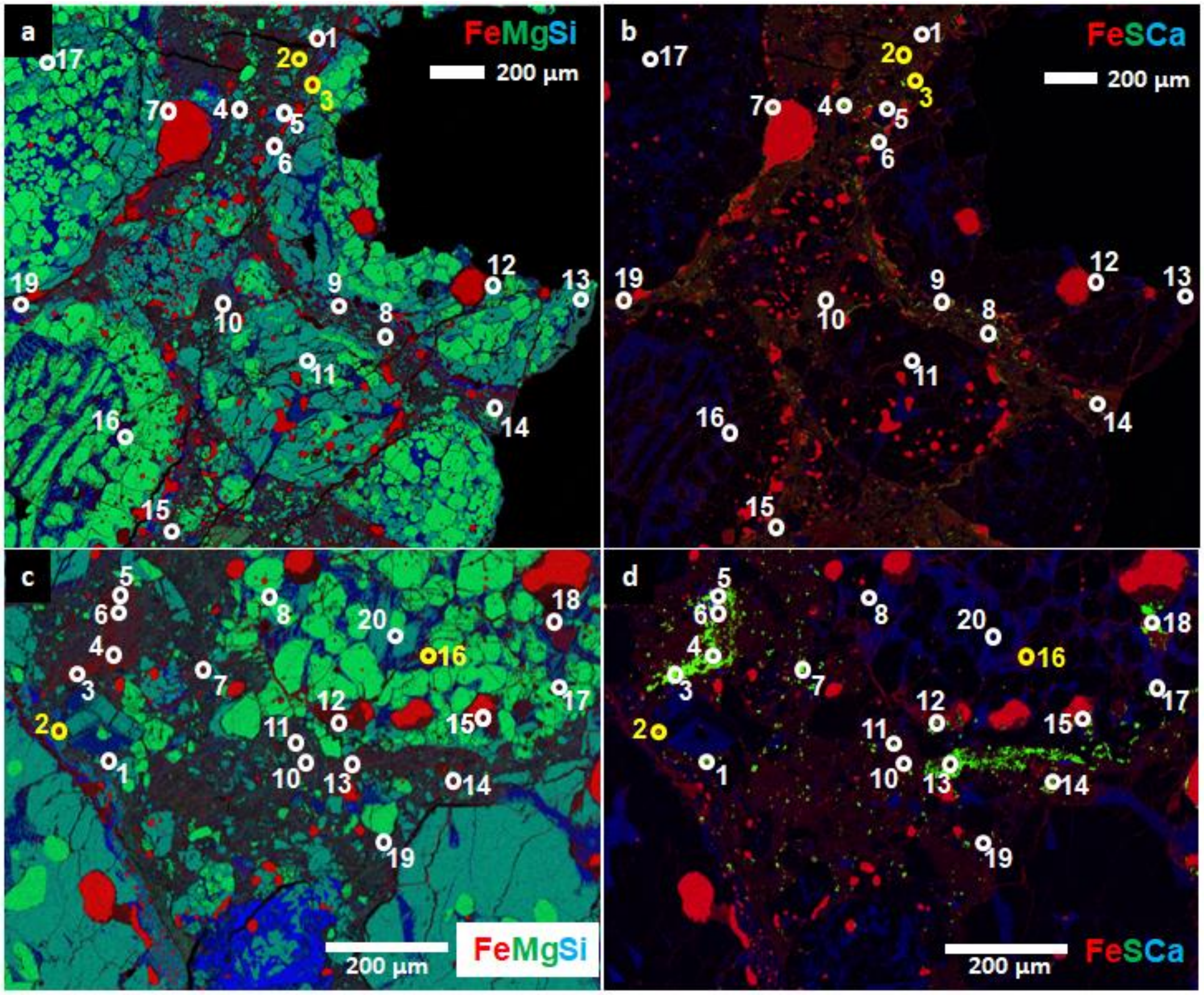

Fig. S9a. *Composite X-ray intensity maps for 2 areas in GRA 95229 that show micro-scale domains rich in elemental sulfur and thiol in their XANES spectra. a and b - Region 4, c and d - region 8; a and c are composites of Fe Kα with Mg Kα and Si Kα intensity maps; b and d are composites of Fe Kα with S Kα and Ca Kα intensity maps; color coding is red for Fe, green for Mg and S, and blue for Ca and Si; numbered white and yellow dots designate XANES analysis spots of Figure S5a; yellow dots indicate the presence of elemental sulfur.*

The CM2 CC Murchison fell on September 28, 1969 near Murchison, Australia, producing a shower of meteorites with a combined mass of 100 kg (*68*, *69*). Murchison is generally characterized by small chondrules and refractory inclusions, abundant fine-grained matrix and hydrated minerals. The dark, fine-grained clastic matrix that accounts for 76 vol% of Murchison (*70*) is dominantly composed of phyllosilicates such as saponite and serpentine (*71*). It contains fragments of chondrules, calcium-aluminum-rich inclusions, spinel, tochilinite, sulfide, and rare metal as inclusions in the matrix (Figure 9b; *71*, *72*). Furthermore, light xenolithic clasts of higher petrologic grade occur in Murchison (*73*, *74*). Murchison is regarded as one of the least altered CMs and hosts pre-solar components (*75*).

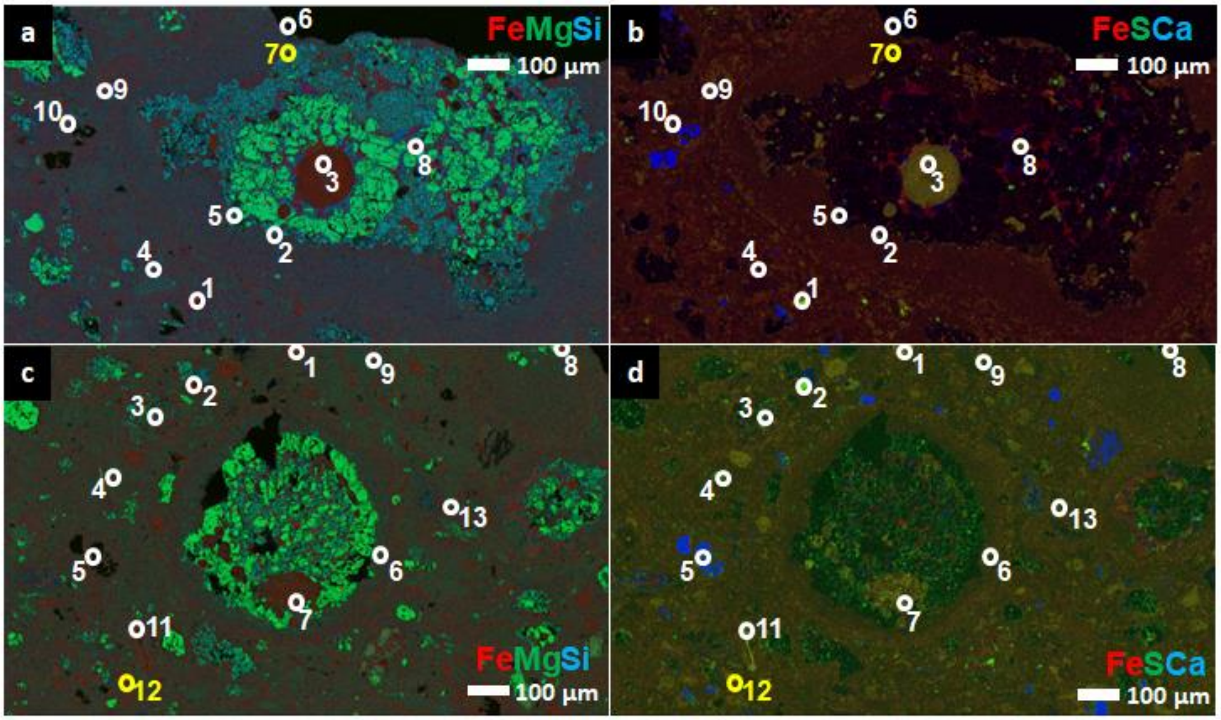

Fig. S9b. *Composite X-ray intensity maps for 2 areas in Murchison that show micro-scale domains rich in elemental sulfur and thiol in their XANES spectra. a and b - region 3, c and d - region 4; a and c are composites of Fe Kα with Mg Kα and Si Kα intensity maps; b and d are composites of Fe Kα with S Kα and Ca Kα intensity maps; color coding is red for Fe, green for Mg and S, and blue for Ca and Si; numbered white and yellow dots designate XANES analysis spots of Figure S5b; yellow dots indicate the presence of elemental sulfur.*

Sulfur is relatively enriched in the inter-clast, fine-grained groundmass of GRA 95229 compared to most clast inclusions. Relatively fewer sulfide regions occur in accreted dust rims on chondrules compared to the groundmass. Sulfur may also occur as altered sulfide components of chondrule fragments. Sulfur speciation appears to be dependent on local alteration conditions and not tied to petrographic context. For example, altered sulfide grains associated with chondrules can show sulfate signatures (Figures S5, S9], while no general correlation is apparent among sulfur-bearing grains in the groundmass and accreted dust rims (Figures S5, S9).

D. Isotopic Data.

D1. Isotopic results on the thiosulfate Grain A0070_TSul#1. The S and O isotopic compositions of the sulfate grain A0070_TSul#1 acquired through an 'isotope' analysis (rastered on the grain) is listed in Table S2.

Table S2. Isotopic Compositions of the Ryugu Grain A0070_Sul

**With reference to**	**δ^{17}O (1σ)**	**δ^{18}O (1σ)**	**Δ^{17}O (1σ)**
San Carlos Olivine*	-11.0±4.3	-7.8±2.3	-7.0±4.9
Eagle Station Olivine	-7.3±3.3	-9.9±1.4	-2.2±3.6
	**δ^{33}S (1σ)**	**δ^{34}S (1σ)**	**Δ^{33}S (1σ)**
TNR14*	-0.1±1.6	9.8±0.9	5.0±1.8
EGT17	-3.1±2.9	-10.4±0.9	2.2±3.0

**delta values plotted in Figure 2 (Main Text)*

D2. Imaging Analysis on the thiosulfate Grain A0070_TSul#1. We measured about 100k counts/sec of ^{32}S on gypsum. On the thiosulfate grain A0070_TSul#1 (with a raster of 8 x 8 µm^2 centered on the grain; Figure S10), the count rate is about 20 times less. The sulfur ratios maps (Figure S10) after correcting for image drift, deadtime and quasi-simultaneous arrival (using a 1 pA current for a semi-quantitative correction using LIMAGE) show homogeneous distribution of S isotopes. We do not report the S isotopes measured during the imaging mode owing to large errors and not using well-characterized organic sulfur standards. A0070_TSul#1 shows C^- and CN^- ion signals. We suspect that the thiol-rich coating is likely methionine (which has N in its structure). The δ^{13}C and δ^{15}N isotopic values for the methionine coating (normalized to the natural ratios, ^{13}C/^{12}C = 0.0112; ^{15}N/^{14}N = 0.003676) are -99 ± 1 ‰ (2σ) and +62.3 ± 2.0 ‰ (2σ), respectively. Owing to the carbon coat that was applied for prior EPMA analysis, the C isotope composition from these images should be used with caution.

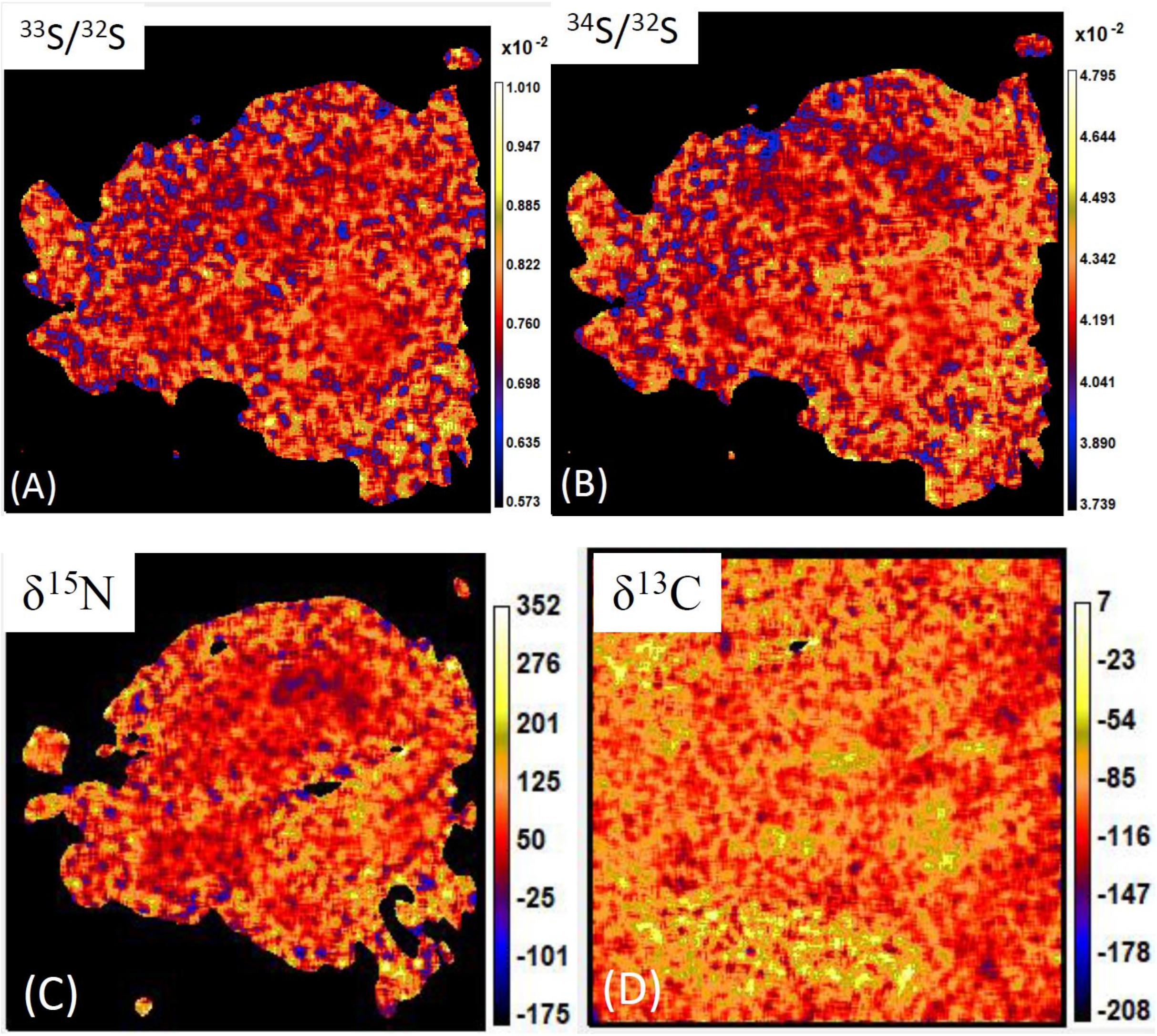

Fig. S10. *The homogeneous distribution of $^{33}S/^{32}S$ and $^{34}S/^{32}S$ isotopic ratios as well as $\delta^{15}N$ and $\delta^{13}C$ image of the thiosulfate grain in Ryugu particle AA0070. The grain coating is N-anomalous.*

REFERENCES AND NOTES

1. K. Kitazato, R. E. Milliken, T. Iwata, M. Abe, M. Ohtake, S. Matsuura, T. Arai, Y. Nakauchi, T. Nakamura, M. Matsuoka, H. Senshu, N. Hirata, T. Hiroi, C. Pilorget, R. Brunetto, F. Poulet, L. Riu, J. P. Bibring, D. Takir, D. L. Domingue, F. Vilas, M. A. Barucci, D. Perna, E. Palomba, A. Galiano, K. Tsumura, T. Osawa, M. Komatsu, A. Nakato, T. Arai, N. Takato, T. Matsunaga, Y. Takagi, K. Matsumoto, T. Kouyama, Y. Yokota, E. Tatsumi, N. Sakatani, Y. Yamamoto, T. Okada, S. Sugita, R. Honda, T. Morota, S. Kameda, H. Sawada, C. Honda, M. Yamada, H. Suzuki, K. Yoshioka, M. Hayakawa, K. Ogawa, Y. Cho, K. Shirai, Y. Shimaki, N. Hirata, A. Yamaguchi, N. Ogawa, F. Terui, T. Yamaguchi, Y. Takei, T. Saiki, S. Nakazawa, S. Tanaka, M. Yoshikawa, S. Watanabe, Y. Tsuda, The surface composition of asteroid 162173 Ryugu from Hayabusa2 near-infrared spectroscopy. *Science* **364**, 272–275 (2019).

2. K. Kitazato, R. E. Milliken, T. Iwata, M. Abe, M. Ohtake, S. Matsuura, Y. Takagi, T. Nakamura, T. Hiroi, M. Matsuoka, L. Riu, Y. Nakauchi, K. Tsumura, T. Arai, H. Senshu, N. Hirata, M. A. Barucci, R. Brunetto, C. Pilorget, F. Poulet, J.-P. Bibring, D. L. Domingue, F. Vilas, D. Takir, E. Palomba, A. Galiano, D. Perna, T. Osawa, M. Komatsu, A. Nakato, T. Arai, N. Takato, T. Matsunaga, M. Arakawa, T. Saiki, K. Wada, T. Kadono, H. Imamura, H. Yano, K. Shirai, M. Hayakawa, C. Okamoto, H. Sawada, K. Ogawa, Y. Iijima, S. Sugita, R. Honda, T. Morota, S. Kameda, E. Tatsumi, Y. Cho, K. Yoshioka, Y. Yokota, N. Sakatani, M. Yamada, T. Kouyama, H. Suzuki, C. Honda, N. Namiki, T. Mizuno, K. Matsumoto, H. Noda, Y. Ishihara, R. Yamada, K. Yamamoto, F. Yoshida, S. Abe, A. Higuchi, Y. Yamamoto, T. Okada, Y. Shimaki, R. Noguchi, A. Miura, N. Hirata, S. Tachibana, H. Yabuta, M. Ishiguro, H. Ikeda, H. Takeuchi, T. Shimada, O. Mori, S. Hosoda, R. Tsukizaki, S. Soldini, M. Ozaki, F. Terui, N. Ogawa, Y. Mimasu, G. Ono, K. Yoshikawa, C. Hirose, A. Fujii, T. Takahashi, S. Kikuchi, Y. Takei, T. Yamaguchi, S. Nakazawa, S. Tanaka, M. Yoshikawa, S. Watanabe, Y. Tsuda, Thermally altered subsurface material of asteroid (162173) Ryugu. *Nat. Astron.* **5**, 246–250 (2021).

3. C. D. K. Herd, Analyzing asteroid Ryugu. *Science* **379**, 784–785 (2023).

4. T. Okada, T. Fukuhara, S. Tanaka, M. Taguchi, T. Arai, H. Senshu, N. Sakatani, Y. Shimaki, H. Demura, Y. Ogawa, K. Suko, T. Sekiguchi, T. Kouyama, J. Takita, T. Matsunaga, T. Imamura, T. Wada, S. Hasegawa, J. Helbert, T. G. Müller, A. Hagermann, J. Biele, M. Grott, M. Hamm,

M. Delbo, N. Hirata, N. Hirata, Y. Yamamoto, S. Sugita, N. Namiki, K. Kitazato, M. Arakawa, S. Tachibana, H. Ikeda, M. Ishiguro, K. Wada, C. Honda, R. Honda, Y. Ishihara, K. Matsumoto, M. Matsuoka, T. Michikami, A. Miura, T. Morota, H. Noda, R. Noguchi, K. Ogawa, K. Shirai, E. Tatsumi, H. Yabuta, Y. Yokota, M. Yamada, M. Abe, M. Hayakawa, T. Iwata, M. Ozaki, H. Yano, S. Hosoda, O. Mori, H. Sawada, T. Shimada, H. Takeuchi, R. Tsukizaki, A. Fujii, C. Hirose, S. Kikuchi, Y. Mimasu, N. Ogawa, G. Ono, T. Takahashi, Y. Takei, T. Yamaguchi, K. Yoshikawa, F. Terui, T. Saiki, S. Nakazawa, M. Yoshikawa, S. Watanabe, Y. Tsuda, Highly porous nature of a primitive asteroid revealed by thermal imaging. *Nature* **579**, 518–522 (2020).

5. M. Grott, J. Knollenberg, M. Hamm, K. Ogawa, R. Jaumann, K. A. Otto, M. Delbo, P. Michel, J. Biele, W. Neumann, M. Knapmeyer, E. Kührt, H. Senshu, T. Okada, J. Helbert, A. Maturilli, N. Müller, A. Hagermann, N. Sakatani, S. Tanaka, T. Arai, S. Mottola, S. Tachibana, I. Pelivan, L. Drube, J.-B. Vincent, H. Yano, C. Pilorget, K. D. Matz, N. Schmitz, A. Koncz, S. E. Schröder, F. Trauthan, M. Schlotterer, C. Krause, T.-M. Ho, A. Moussi-Soffys, Low thermal conductivity boulder with high porosity identified on C-type asteroid (162173) *Ryugu. Nat. Astron.* **3**, 971–976 (2019).

6. H. Yabuta, G. D. Cody, C. Engrand, Y. Kebukawa, B. de Gregorio, L. Bonal, L. Remusat, R. Stroud, E. Quirico, L. Nittler, M. Hashiguchi, M. Komatsu, T. Okumura, J. Mathurin, E. Dartois, J. Duprat, Y. Takahashi, Y. Takeichi, D. Kilcoyne, S. Yamashita, A. Dazzi, A. Deniset-Besseau, S. Sandford, Z. Martins, Y. Tamenori, T. Ohigashi, H. Suga, D. Wakabayashi, M. Verdier-Paoletti, S. Mostefaoui, G. Montagnac, J. Barosch, K. Kamide, M. Shigenaka, L. Bejach, M. Matsumoto, Y. Enokido, T. Noguchi, H. Yurimoto, T. Nakamura, R. Okazaki, H. Naraoka, K. Sakamoto, H. C. Connolly Jr., D. S. Lauretta, M. Abe, T. Okada, T. Yada, M. Nishimura, K. Yogata, A. Nakato, M. Yoshitake, A. Iwamae, S. Furuya, K. Hatakeda, A. Miyazaki, H. Soejima, Y. Hitomi, K. Kumagai, T. Usui, T. Hayashi, D. Yamamoto, R. Fukai, S. Sugita, K. Kitazato, N. Hirata, R. Honda, T. Morota, E. Tatsumi, N. Sakatani, N. Namiki, K. Matsumoto, R. Noguchi, K. Wada, H. Senshu, K. Ogawa, Y. Yokota, Y. Ishihara, Y. Shimaki, M. Yamada, C. Honda, T. Michikami, M. Matsuoka, N. Hirata, M. Arakawa, C. Okamoto, M. Ishiguro, R. Jaumann, J. P. Bibring, M. Grott, S. Schröder, K. Otto, C. Pilorget, N. Schmitz, J. Biele, T. M. Ho, A. Moussi-Soffys, A. Miura, H. Noda, T. Yamada, K. Yoshihara, K. Kawahara, H. Ikeda, Y. Yamamoto, K. Shirai, S. Kikuchi, N. Ogawa, H. Takeuchi, G. Ono, Y. Mimasu, K. Yoshikawa, Y. Takei, A.

Fujii, Y. I. Iijima, S. Nakazawa, S. Hosoda, T. Iwata, M. Hayakawa, H. Sawada, H. Yano, R. Tsukizaki, M. Ozaki, F. Terui, S. Tanaka, M. Fujimoto, M. Yoshikawa, T. Saiki, S. Tachibana, S. I. Watanabe, Y. Tsuda, Macromolecular organic matter in samples of the asteroid (162173) Ryugu. *Science* **379**, 790–803 (2023).

7. T. Yoshimura, Y. Takano, H. Naraoka, T. Koga, D. Araoka, N. O. Ogawa, P. Schmitt-Kopplin, N. Hertkorn, Y. Oba, J. P. Dworkin, J. C. Aponte, T. Yoshikawa, S. Tanaka, N. Ohkouchi, M. Hashiguchi, H. McLain, E. T. Parker, S. Sakai, M. Yamaguchi, T. Suzuki, T. Yokoyama, H. Yurimoto, T. Nakamura, T. Noguchi, R. Okazaki, H. Yabuta, K. Sakamoto, T. Yada, M. Nishimura, A. Nakato, A. Miyazaki, K. Yogata, M. Abe, T. Okada, T. Usui, M. Yoshikawa, T. Saiki, S. Tanaka, F. Terui, S. Nakazawa, S. I. Watanabe, Y. Tsuda, S. Tachibana, Hayabusa2-initial-analysis SOM team, K. Hamase, A. Furusho, K. Fukushima, D. Aoki, D. P. Glavin, H. L. McLain, J. E. Elsila, H. V. Graham, J. M. Eiler, A. Ruf, F. R. Orthous-Daunay, C. Wolters, J. Isa, V. Vuitton, R. Thissen, H. Sugahara, H. Mita, Y. Furukawa, Y. Chikaraishi, M. Morita, M. Onose, F. Kabashima, K. Fujishima, H. Sato, K. Sasaki, K. Kano, S. I. M. Nomura, J. Aoki, T. Yamazaki, Y. Kimura, Chemical evolution of primordial salts and organic sulfur molecules in the asteroid 162173 Ryugu. *Nat. Commun.* **14**, 5284–5295 (2023).

8. P. R. Buseck, X. Hua, Matrices of carbonaceous chondrite meteorites. *Annu. Rev. Earth Planet. Sci.* **21**, 255–305 (1993).

9. S. A. Singerling, A. J. Brearley, Primary iron sulfides in CM and CR carbonaceous chondrites: Insights into nebular processes. *Meteor. Planet. Sci.* **53**, 2078–2106 (2018).

10. S. A. Singerling, A. J. Brearley, Altered primary iron sulfides in CM2 and CR2 carbonaceous chondrites: Insights into parent body processes. *Meteorit. Planet. Sci.* **55**, 496–523 (2020).

11. E. Goresy, H. Yabuki, E. Pernicka, A tentative alphabet for the EH chondrites. *Antarct. Meteor. Res.* **1**, 65–101 (1988).

12. M. Shimizu, H. Yoshida, J. A. Mandarino, The new mineral species keilite, (Fe,Mg)S, the IRON-DOMINANT analogue of niningerite. *Canadian Mineral.* **40**, 1687–1692 (2002).

13. E. M. Levin, S. L. Bud’ko, J. D. Mao, Y. Huang, K. Schmidt-Rohr, Effect of magnetic particles on NMR spectra of murchison meteorite organic matter and a polymer-based model system. *Solid State Nucl. Magn. Reson.* **31**, 63–71 (2007).

14. M. R. Lee, The petrography, mineralogy and origins of calcium sulphate within the Cold Bokkeveld CM carbonaceous chondrite. *Meteoritics* **28**, 53–62 (1993).

15. M. Zolensky, Mineralogy and composition of matrix and chondrule rims in carbonaceous chondrites. *Geochim. Cosmochim. Acta* **57**, 3123–3148 (1993).

16. G. W. Cooper, M. H. Thiemens, T. L. Jackson, S. Chang, Sulfur and hydrogen isotope anomalies in meteorite sulfonic acids. *Science* **277**, 1072–1074 (1997).

17. L. Remusat, S. Derenne, F. Robert, H. Knicker, New pyrolytic and spectroscopic data on Orgueil and Murchison insoluble organic matter: A different origin than soluble. *Geochim. Cosmochim. Acta* **69**, 3919–3932 (2005).

18. H. Yabuta, L. B. Williams, G. D. Cody, C. M. O’D Alexander, S. Pizzarello, The insoluble carbonaceous material of CM chondrites: A possible source of discrete organic compounds under hydrothermal conditions. *Meteorit. Planet. Sci.* **42**, 37–48 (2007).

19. F. R. Orthous-Daunay, E. Quirico, L. Lemelle, P. Beck, V. deAndrade, A. Simionovici, S. Derenne, Speciation of sulfur in the insoluble organic matter from carbonaceous chondrites by XANES spectroscopy. *Earth Planet. Sci. Lett.* **300**, 321–328 (2010).

20. M. Bose, R. A. Root, S. Pizzarello, A XANES and Raman investigation of sulfur speciation and structural order in Murchison and Allende meteorites. *Meteorit. Planet. Sci.* **52**, 546–559 (2017).

21. C. M. O’D Alexander, J. G. Wynn, R. Bowden, Sulfur abundances and isotopic compositions in bulk carbonaceous chondrites and insoluble organic material: Clues to elemental and isotopic fractionations of volatile chalcophiles. *Meteorit. Planet. Sci.* **57**, 334–351 (2021).

22. M. C. Liu, K. A. McCain, N. Matsuda, A. Yamaguchi, M. Kimura, N. Tomioka, M. Ito, M. Uesugi, N. Imae, N. Shirai, T. Ohigashi, R. C. Greenwood, K. Uesugi, A. Nakato, K. Yogata, H.

Yuzawa, Y. Kodama, K. Hirahara, I. Sakurai, I. Okada, Y. Karouji, S. Nakazawa, T. Okada, T. Saiki, S. Tanaka, F. Terui, M. Yoshikawa, A. Miyazaki, M. Nishimura, T. Yada, M. Abe, T. Usui, S. I. Watanabe, Y. Tsuda, Incorporation of ^{16}O-rich anhydrous silicates in the protolith of highly hydrated asteroid Ryugu. *Nat. Astron.* **6**, 1172–1177 (2022).

23. N. Kawasaki, K. Nagashima, N. Sakamoto, T. Matsumoto, K. I. Bajo, S. Wada, Y. Igami, A. Miyake, T. Noguchi, D. Yamamoto, S. S. Russell, Y. Abe, J. Aléon, C. M. O'D Alexander, S. Amari, Y. Amelin, M. Bizzarro, A. Bouvier, R. W. Carlson, M. Chaussidon, B. G. Choi, N. Dauphas, A. M. Davis, T. di Rocco, W. Fujiya, R. Fukai, I. Gautam, M. K. Haba, Y. Hibiya, H. Hidaka, H. Homma, P. Hoppe, G. R. Huss, K. Ichida, T. Iizuka, T. R. Ireland, A. Ishikawa, M. Ito, S. Itoh, N. T. Kita, K. Kitajima, T. Kleine, S. Komatani, A. N. Krot, M. C. Liu, Y. Masuda, K. D. McKeegan, M. Morita, K. Motomura, F. Moynier, I. Nakai, A. Nguyen, L. Nittler, M. Onose, A. Pack, C. Park, L. Piani, L. Qin, M. Schönbächler, L. Tafla, H. Tang, K. Terada, Y. Terada, T. Usui, M. Wadhwa, R. J. Walker, K. Yamashita, Q. Z. Yin, T. Yokoyama, S. Yoneda, E. D. Young, H. Yui, A. C. Zhang, T. Nakamura, H. Naraoka, R. Okazaki, K. Sakamoto, H. Yabuta, M. Abe, A. Miyazaki, A. Nakato, M. Nishimura, T. Okada, T. Yada, K. Yogata, S. Nakazawa, T. Saiki, S. Tanaka, F. Terui, Y. Tsuda, S. I. Watanabe, M. Yoshikawa, S. Tachibana, H. Yurimoto, Oxygen isotopes of anhydrous primary minerals show kinship between asteroid Ryugu and Comet *81P/Wild2. Sci. Adv.* **8**, eade2067 (2022).

24. D. Nakashima, T. Nakamura, M. Zhang, N. T. Kita, T. Mikouchi, H. Yoshida, Y. Enokido, T. Morita, M. Kikuiri, K. Amano, E. Kagawa, T. Yada, M. Nishimura, A. Nakato, A. Miyazaki, K. Yogata, M. Abe, T. Okada, T. Usui, M. Yoshikawa, T. Saiki, S. Tanaka, S. Nakazawa, F. Terui, H. Yurimoto, T. Noguchi, H. Yabuta, H. Naraoka, R. Okazaki, K. Sakamoto, S. I. Watanabe, S. Tachibana, Y. Tsuda, Chondrule-like objects and Ca-Al-rich Inclusions in Ryugu may potentially be the oldest solar system materials. *Nat. Commun.* **14**, 532 (2023).

25. L. G. Vacher, R. C. Ogliore, C. Jones, N. Liu, D. A. Fike, Cosmic symplectite recorded irradiation by nearby massive stars in the solar system's parent molecular cloud. *Geochim. Cosmochim. Acta* **309**, 135–150 (2021).

26. V. K. Rai, M. H. Thiemens, Mass independently fractionated sulfur components in chondrites. *Geochim. Cosmochim. Acta* **71**, 1341–1354 (2007).

27. E. S. Bullock, K. V. McKeegan, M. Gounelle, M. M. Grady, S. S. Russell, Sulfur isotopic composition of Fe-Ni sulfide grains in CI and CM carbonaceous chondrites. *Meteorit. Planet. Sci.* **45**, 885–898 (2010).

28. J. Farquhar, J. Savarino, S. Airieau, M. H. Thiemens, Observation of wavelength-sensitive mass-independent sulfur isotope effects during SO_2 photolysis: Implications for the early atmosphere. *J. Geophys. Res. Planets* **106**, 32829–32839 (2001).

29. A. A. Pavlov, J. F. Kasting, Mass-independent fractionation of sulfur isotopes in archean sediments: Strong evidence for an anoxic archean atmosphere. *Astrobiology* **2**, 27–41 (2002).

30. K. A. Mc Cain, N. Matsuda, M.-C. Liu, K. D. Mc Keegan, A. Yamaguchi, M. Kimura, N. Tomioka, M. Ito, N. Imae, M. Uesugi, N. Shirai, T. Ohigashi, R. C. Greenwood, K. Uesugi, A. Nakato, K. Yogata, H. Yuzawa, Y. Kodama, K. Hirahara, I. Sakurai, I. Okada, Y. Karouji, S. Nakazawa, T. Okada, T. Saiki, S. Tanaka, F. Terui, M. Yoshikawa, A. Miyazaki, M. Nishimura, T. Yada, M. Abe, T. Usui, S.-I. Watanabe, Y. Tsuda, Early fluid activity on Ryugu inferred by isotopic analyses of carbonates and magnetite. *Nat. Astron.* **7**, 309–317 (2023).

31. S. J. Desch, A. Kalyaan, C. M. O'D Alexander, The effect of Jupiter's formation on the distribution of refractory elements and inclusions in meteorites. *Astrophys. J. Supplement. Ser.* **238**, 11–42 (2018).

32. S. B. Charnley, S. D. Rodgers, The end of interstellar chemistry as the origin of nitrogen in comets and meteorites. *Astrophys. J.* **569**, L133-L137 (2002).

33. N. Sakamoto, Y. Seto, S. Itoh, K. Kuramoto, K. Fujino, K. Nagashima, A. N. Krot, H. Yurimoto Remnants of the early solar system water enriched in heavy oxygen isotopes. *Science* **317**, 231–233 (2007).

34. H. Gamsjager, R. K. Murmann. “Oxygen-18 exchange studies of aqua- and oxo-ions” in *Advances in Inorganic and Bioinorganic Mechanisms,* A. G. Skyes, Ed., (Academic Press, 1983), vol. 2, pp. 317–380.

35. M. A. Velbel, E. K. Tonui, M. E. Zolensky, Replacement of olivine by serpentine in the carbonaceous chondrite nogoya (CM2). *Geochim. Cosmochim. Acta* **87**, 117–135 (2012).

36. C. Connolly, K. Muehlenbachs, Contrasting oxygen diffusion in nepheline, diopside and other silicates and their relevance to isotopic systematics in meteorites. *Geochim. Cosmochim. Acta* **52**, 1585–1591 (1988).

37. D. R. Van Stempvoort, H. R. Krouse, “Controls of δ^{18} O in sulfate: Review of experimental data and application to specific environments” in *Environmental Geochemistry of Sulfide Oxidation*, C. N. Alpers, D. W. Blowes, Eds. (ACS Publications, 1993), Chapter 29, pp. 446–480.

38. M. Ito, N. Tomioka, M. Uesugi, A. Yamaguchi, N. Shirai, T. Ohigashi, M. C. Liu, R. C. Greenwood, M. Kimura, N. Imae, K. Uesugi, A. Nakato, K. Yogata, H. Yuzawa, Y. Kodama, A. Tsuchiyama, M. Yasutake, R. Findlay, I. A. Franchi, J. A. Malley, K. A. McCain, N. Matsuda, K. D. McKeegan, K. Hirahara, A. Takeuchi, S. Sekimoto, I. Sakurai, I. Okada, Y. Karouji, M. Arakawa, A. Fujii, M. Fujimoto, M. Hayakawa, N. Hirata, N. Hirata, R. Honda, C. Honda, S. Hosoda, Y. I. Iijima, H. Ikeda, M. Ishiguro, Y. Ishihara, T. Iwata, K. Kawahara, S. Kikuchi, K. Kitazato, K. Matsumoto, M. Matsuoka, T. Michikami, Y. Mimasu, A. Miura, O. Mori, T. Morota, S. Nakazawa, N. Namiki, H. Noda, R. Noguchi, N. Ogawa, K. Ogawa, T. Okada, C. Okamoto, G. Ono, M. Ozaki, T. Saiki, N. Sakatani, H. Sawada, H. Senshu, Y. Shimaki, K. Shirai, S. Sugita, Y. Takei, H. Takeuchi, S. Tanaka, E. Tatsumi, F. Terui, R. Tsukizaki, K. Wada, M. Yamada, T. Yamada, Y. Yamamoto, H. Yano, Y. Yokota, K. Yoshihara, M. Yoshikawa, K. Yoshikawa, R. Fukai, S. Furuya, K. Hatakeda, T. Hayashi, Y. Hitomi, K. Kumagai, A. Miyazaki, M. Nishimura, H. Soejima, A. Iwamae, D. Yamamoto, M. Yoshitake, T. Yada, M. Abe, T. Usui, S. I. Watanabe, Y. Tsuda, A pristine record of outer Solar System materials from asteroid Ryugu’s returned sample. *Nat. Astron.* **6**, 1163–1171 (2022).

39. T. Nakamura, M. Matsumoto, K. Amano, Y. Enokido, M. E. Zolensky, T. Mikouchi, H. Genda, S. Tanaka, M. Y. Zolotov, K. Kurosawa, S. Wakita, R. Hyodo, H. Nagano, D. Nakashima, Y.

Takahashi, Y. Fujioka, M. Kikuiri, E. Kagawa, M. Matsuoka, A. J. Brearley, A. Tsuchiyama, M. Uesugi, J. Matsuno, Y. Kimura, M. Sato, R. E. Milliken, E. Tatsumi, S. Sugita, T. Hiroi, K. Kitazato, D. Brownlee, D. J. Joswiak, M. Takahashi, K. Ninomiya, T. Takahashi, T. Osawa, K. Terada, F. E. Brenker, B. J. Tkalcec, L. Vincze, R. Brunetto, A. Aléon-Toppani, Q. H. S. Chan, M. Roskosz, J.-C. Viennet, P. Beck, E. E. Alp, T. Michikami, Y. Nagaashi, T. Tsuji, Y. Ino, J. Martinez, J. Han, A. Dolocan, R. J. Bodnar, M. Tanaka, H. Yoshida, K. Sugiyama, A. J. King, K. Fukushi, H. Suga, S. Yamashita, T. Kawai, K. Inoue, A. Nakato, T. Noguchi, F. Vilas, A. R. Hendrix, C. Jaramillo-Correa, D. L. Domingue, G. Dominguez, Z. Gainsforth, C. Engrand, J. Duprat, S. S. Russell, E. Bonato, C. Ma, T. Kawamoto, T. Wada, S. Watanabe, R. Endo, S. Enju, L. Riu, S. Rubino, P. Tack, S. Takeshita, Y. Takeichi, A. Takeuchi, A. Takigawa, D. Takir, T. Tanigaki, A. Taniguchi, K. Tsukamoto, T. Yagi, S. Yamada, K. Yamamoto, Y. Yamashita, M. Yasutake, K. Uesugi, I. Umegaki, I. Chiu, T. Ishizaki, S. Okumura, E. Palomba, C. Pilorget, S. M. Potin, A. Alasli, S. Anada, Y. Araki, N. Sakatani, C. Schultz, O. Sekizawa, S. D. Sitzman, K. Sugiura, M. Sun, E. Dartois, E. De Pauw, Z. Dionnet, Z. Djouadi, G. Falkenberg, R. Fujita, T. Fukuma, I. R. Gearba, K. Hagiya, M. Y. Hu, T. Kato, T. Kawamura, M. Kimura, M. K. Kubo, F. Langenhorst, C. Lantz, B. Lavina, M. Lindner, J. Zhao, B. Vekemans, D. Baklouti, B. Bazi, F. Borondics, S. Nagasawa, G. Nishiyama, K. Nitta, J. Mathurin, T. Matsumoto, I. Mitsukawa, H. Miura, A. Miyake, Y. Miyake, H. Yurimoto, R. Okazaki, H. Yabuta, H. Naraoka, K. Sakamoto, S. Tachibana, H. C. Connolly Jr., D. S. Lauretta, M. Yoshitake, M. Yoshikawa, K. Yoshikawa, K. Yoshihara, Y. Yokota, K. Yogata, H. Yano, Y. Yamamoto, D. Yamamoto, M. Yamada, T. Yamada, T. Yada, K. Wada, T. Usui, R. Tsukizaki, F. Terui, H. Takeuchi, Y. Takei, A. Iwamae, H. Soejima, K. Shirai, Y. Shimaki, H. Senshu, H. Sawada, T. Saiki, M. Ozaki, G. Ono, T. Okada, N. Ogawa, K. Ogawa, R. Noguchi, H. Noda, M. Nishimura, N. Namiki, S. Nakazawa, T. Morota, A. Miyazaki, A. Miura, Y. Mimasu, K. Matsumoto, K. Kumagai, T. Kouyama, S. Kikuchi, K. Kawahara, S. Kameda, T. Iwata, Y. Ishihara, M. Ishiguro, H. Ikeda, S. Hosoda, R. Honda, C. Honda, Y. Hitomi, N. Hirata, N. Hirata, T. Hayashi, M. Hayakawa, K. Hatakeda, S. Furuya, R. Fukai, A. Fujii, Y. Cho, M. Arakawa, M. Abe, S. Watanabe, Y. Tsuda, Formation and evolution of carbonaceous asteroid Ryugu: Direct evidence from returned samples. *Science* **379**, eabn8671 (2022).

40. H. Naraoka, Y. Takano, J. P. Dworkin, Y. Oba, K. Hamase, A. Furusho, N. O. Ogawa, M. Hashiguchi, K. Fukushima, D. Aoki, P. Schmitt-Kopplin, J. C. Aponte, E. T. Parker, D. P. Glavin, H. L. M. Lain, J. E. Elsila, H. V. Graham, J. M. Eiler, F.-R. Orthous-Daunay, C. Wolters, J. Isa, V. Vuitton, R. Thissen, S. Sakai, T. Yoshimura, T. Koga, N. Ohkouchi, Y. Chikaraishi, H. Sugahara, H. Mita, Y. Furukawa, N. Hertkorn, A. Ruf, H. Yurimoto, T. Nakamura, T. Noguchi, R. Okazaki, H. Yabuta, K. Sakamoto, S. Tachibana, H. C. Connolly Jr., D. S. Lauretta, M. Abe, T. Yada, M. Nishimura, K. Yogata, A. Nakato, M. Yoshitake, A. Suzuki, A. Miyazaki, S. Furuya, K. Hatakeda, H. Soejima, Y. Hitomi, K. Kumagai, T. Usui, T. Hayashi, D. Yamamoto, R. Fukai, K. Kitazato, S. Sugita, N. Namiki, M. Arakawa, H. Ikeda, M. Ishiguro, N. Hirata, K. Wada, Y. Ishihara, R. Noguchi, T. Morota, N. Sakatani, K. Matsumoto, H. Senshu, R. Honda, E. Tatsumi, Y. Yokota, C. Honda, T. Michikami, M. Matsuoka, A. Miura, H. Noda, T. Yamada, K. Yoshihara, K. Kawahara, M. Ozaki, Y.-I. Iijima, H. Yano, M. Hayakawa, T. Iwata, R. Tsukizaki, H. Sawada, S. Hosoda, K. Ogawa, C. Okamoto, N. Hirata, K. Shirai, Y. Shimaki, M. Yamada, T. Okada, Y. Yamamoto, H. Takeuchi, A. Fujii, Y. Takei, K. Yoshikawa, Y. Mimasu, G. Ono, N. Ogawa, S. Kikuchi, S. Nakazawa, F. Terui, S. Tanaka, T. Saiki, M. Yoshikawa, S.-I. Watanabe, Y. Tsuda, Soluble organic molecules in samples of the carbonaceous asteroid (162173) Ryugu. *Science* **379**, 6634 (2023).

41. L. R. Nittler, J. Barosch, K. Burgess, R. M. Stroud, J. Wang, H. Yabuta, Y. Enokido, M. Matsumoto, T. Nakamura, Y. Kebukawa, S. Yamashita, Y. Takahashi, L. Bejach, L. Bonal, G. D. Cody, E. Dartois, A. Dazzi, B. de Gregorio, A. Deniset-Besseau, J. Duprat, C. Engrand, M. Hashiguchi, A. L. D. Kilcoyne, M. Komatsu, Z. Martins, J. Mathurin, G. Montagnac, S. Mostefaoui, T. Okumura, E. Quirico, L. Remusat, S. Sandford, M. Shigenaka, H. Suga, Y. Takeichi, Y. Tamenori, M. Verdier-Paoletti, D. Wakabayashi, M. Abe, K. Kamide, A. Miyazaki, A. Nakato, S. Nakazawa, M. Nishimura, T. Okada, T. Saiki, S. Tanaka, F. Terui, T. Usui, T. Yada, K. Yogata, M. Yoshikawa, H. Yurimoto, T. Noguchi, R. Okazaki, H. Naraoka, K. Sakamoto, S. Tachibana, S. I. Watanabe, Y. Tsuda, Microscale hydrogen, carbon, and nitrogen isotopic diversity of organic matter in asteroid Ryugu. *Earth Planet. Sci. Lett.* **637**, 118719 (2024).

42. H. Busemann, A. F. Young, C. M. O'D Alexander, P. Hoppe, S. Mukhopadhyay, L. R. Nittler, Interstellar chemistry recorded in organic matter from primitive meteorites. *Science* **312**, 727–730 (2006).

43. L. Remusat, F. Robert, A. Meibom, S. Mostefaoui, O. Delpoux, L. Binet, D. Gourier, S. Derenne, Proto-planetary disk chemistry recorded by D-rich organic radicals in carbonaceous chondrites. *Astrophys. J.* **698**, 2087–2092 (2009).

44. C. Pilorget, T. Okada, V. Hamm, R. Brunetto, T. Yada, D. Loizeau, L. Riu, T. Usui, A. Moussi-Soffys, K. Hatakeda, A. Nakato, K. Yogata, M. Abe, A. Aléon-Toppani, J. Carter, M. Chaigneau, B. Crane, B. Gondet, K. Kumagai, Y. Langevin, C. Lantz, T. le Pivert-Jolivet, G. Lequertier, L. Lourit, A. Miyazaki, M. Nishimura, F. Poulet, M. Arakawa, N. Hirata, K. Kitazato, S. Nakazawa, N. Namiki, T. Saiki, S. Sugita, S. Tachibana, S. Tanaka, M. Yoshikawa, Y. Tsuda, S. Watanabe, J. P. Bibring, First compositional analysis of Ryugu samples by the micromega hyperspectral microscope. *Nat. Astron.* **6**, 221–225 (2022).

45. M. Roskosz, P. Beck, J. C. Viennet, T. Nakamura, B. Lavina, M. Y. Hu, J. Zhao, E. E. Alp, Y. Takahashi, T. Morita, K. Amano, H. Yurimoto, T. Noguchi, R. Okazaki, H. Yabuta, H. Naraoka, K. Sakamoto, S. Tachibana, T. Yada, M. Nishimura, A. Nakato, A. Miyazaki, K. Yogata, M. Abe, T. Okada, T. Usui, M. Yoshikawa, T. Saiki, S. Tanaka, F. Terui, S. Nakazawa, S. I. Watanabe, Y. Tsuda, The iron oxidation state of Ryugu samples. *Meteorit. Planet. Sci.* **1–22**, https://doi.org/10.1111/maps.14098 (2023).

46. J. C. Aponte, J. P. Dworkin, D. P. Glavin, J. E. Elsila, E. T. Parker, H. L. McLain, H. Naraoka, R. Okazaki, Y. Takano, S. Tachibana, G. Dong, S. S. Zeichner, J. M. Eiler, H. Yurimoto, T. Nakamura, H. Yabuta, F. Terui, T. Noguchi, K. Sakamoto, T. Yada, M. Nishimura, A. Nakato, A. Miyazaki, K. Yogata, M. Abe, T. Okada, T. Usui, M. Yoshikawa, T. Saiki, S. Tanaka, S. Nakazawa, Y. Tsuda, S. I. Watanabe, The Hayabusa2-initial-analysis SOM team, The Hayabusa2-initial-analysis core team, PAHs, hydrocarbons, and dimethylsulfides in Asteroid Ryugu samples A0106 and C0107 and the Orgueil (CI1) meteorite. *Earth Planets Space* **75**, 28 (2023).

47. M. Bose, R. Root, Identification of organic and elemental sulfur in the fine-grained matrix of Graves Nunataks GRA 95229, in *Proceedings of the 2018 49th Lunar and Planetary Science Conference* (Lunar and Planetary Institute, 2018), Abstr. 2098.

48. R. A. Root, S. Fathordoobadi, F. Alday, W. Ela, J. Chorover, Microscale speciation of arsenic and iron in ferric-based sorbents subjected to simulated landfill conditions. *Environ. Sci. Technol.* **47**, 12992–13000 (2013).

49. S. M. Webb, SIXpack: A graphical user interface for XAS analysis using IFEFFIT. *Phys. Scr.* **2005**, 1011–1014 (2005).

50. B. Ravel, M. Newville, ATHENA, ARTEMIS, HEPHAESTUS: Data analysis for x-ray absorption spectroscopy using IFEFFIT. *J. Synchrotron Radiat.* **12**, 537–541 (2005).

51. M. Ito, M. Uesugi, H. Naraoka, H. Yabuta, F. Kitajima, H. Mita, Y. Takano, Y. Karouji, T. Yada, Y. Ishibashi, T. Okada, M. Abe, H, C, and N isotopic compositions of hayabusa category 3 organic samples. *Earth Planets Space* **66**, 91–110 (2014).

52. D. A. Fike, C. L. Gammon, W. Ziebis, V. J. Orphan, Micron-scale mapping of sulfur cycling across the oxycline of a cyanobacterial mat: A paired NanoSIMS and CARD-FISH approach. *ISME J.* **2**, 749–759 (2008).

53. Z. Taracsák, D. A. Neave, P. Beaudry, J. Gunnarsson-Robin, R. Burgess, M. Edmonds, S. A. Halldórsson, M.-A. Longpré, S. Ono, E. Ranta, A. Stefánsson, A. V. Turchyn, EIMF, M. E. Hartley, Instrumental mass fractionation during sulfur isotope analysis by secondary ion mass spectrometry in natural and synthetic glasses. *Chem. Geol.* **578**, 120318 (2021).

54. L. Bindi, J. M. Eiler, Y. Guan, L. S. Hollister, G. MacPherson, P. J. Steinhardt, N. Yao, Evidence for the extraterrestrial origin of a natural quasicrystal. *Proc. Natl. Acad. Sci. U.S.A* **109**, 1396–1401 (2012).

55. R. N. Clayton, T. K. Mayeda, Oxygen isotope studies of achondrites. *Geochim. Cosmochim. Acta* **60**, 1999–2017 (1996).

56. A. Pack, R. Tanaka, M. Hering, S. Sengupta, S. Peters, E. Nakamura, The oxygen isotope composition of san carlos olivine on the VSMOW2-SLAP2 Scale. *Rapid Commun. Mass Spectrom.* **30**, 1495–1504 (2016).

57. C. M. Bethke, *Geochemical and Biogeochemical Reaction Modeling* (Cambridge Univ. Press, 2012).

58. J. M. Delany, S. R. Lundeen, The LLNL Thermochemical Database, Lawrence Livermore National Laboratory Report (1990).

59. H. C. Helgeson, Summary and critique of the thermodynamic properties of rock-forming minerals. *Am. J. Sci.* **278A**, 1–229 (1978).

60. H. C. Helgeson, Thermodynamics of hydrothermal systems at elevated temperatures and pressures. *Am. J. Sci.* **267**, 729804 (1969).

61. E.D. Young, S.S. Russell, Oxygen reservoirs in the early solar nebula inferred from an Allende CAI. *Science* **282**, 452-455 (1998).

62. K. Xia, F. Weesner, W. F. Bleam, P. A. Helmke, P. R. Bloom, U. L. Skyllberg, XANES studies of oxidation states of sulfur in aquatic and soil humic substances. *Soil Sci. Soc. Am. J.* **62**, 1240–1246 (1998).

63. J. Davidson, D. L. Schrader, The CR chondrites: Treasure troves from the early solar system. *Elements* **19**, 127–128 (2023).

64. J. N. Grossman, The Meteoritical Bulletin, No. 82, July 1998. *U.S. Geological Survey* 33, A221–A239 (1998).

65. D. L. Schrader, I. A. Franchi, H. C. Connolly Jr., R. C. Greenwood, D. S. Lauretta, J. M. Gibson, The formation and alteration of the renazzo-like carbonaceous chondrites I: Implications of bulk-oxygen isotopic composition. *Geochim. Cosmochim. Acta* **75**, 308–325 (2011).

66. N. M. Abreu, Why is it so difficult to classify renazzo-type (CR) carbonaceous chondrites?–Implications from TEM observations of matrices for the sequences of aqueous alteration. *Geochim. Cosmochim. Acta* **194**, 91–122 (2016).

67. J. Leitner, P. Hoppe, J. Zipfel, The stardust inventories of Graves Nunataks 95229 and Renazzo: Implications for the distribution of presolar grains in CR chondrites, (Abstract #1835). *43rd Lunar and Planetary Science Conference* (Houston TX, 2012).

68. J. F. Lovering, R. W. Le Maitre, B. W. Chappell, Murchison C2 carbonaceous chrondrite and its inorganic composition. *Nat. Phys. Sci.* **230**, 18–20 (1971).

69. L. H. Fuchs, E. Olsen, K. J. Jensen, Mineralogy, mineral-chemistry, and composition of the murchison (C2) meteorite. *Smithsonian Contribut. Earth Sci.* **1–39**, 1–39 (1973).

70. K. Metzler, A. Bischoff, D. Stoffler, Accretionary dust mantles in CM chondrites: Evidence for solar nebula processes. *Geochim. Cosmochim. Acta* **56**, 2873–2897 (1992).

71. A. J. Brearley, R. H. Jones, J. J. Papike, Ed. (1998). Chondritic meteorites. In Planetary Materials (Reviews in Mineralogy), vol. 36, pp. C1.

72. I. D. R. Mackinnon, M. E. Zolensky, Proposed structures for poorly characterized phases in C2M carbonaceous chondrite meteorites. *Nature* **309**, 240–242 (1984).

73. E. J. Olsen, A. M. Davis, I. D. Hutcheon, R. N. Clayton, T. K. Mayeda, L. Grossman, Murchison xenoliths. *Geochim. Cosmochim. Acta* **52**, 1615–1626 (1988).

74. I. Kerraouch, S. Ebert, M. Patzek, A. Bischoff, M. E. Zolensky, A. Pack, P. Schmitt-Kopplin, D. Belhai, A. Bendaoud, L. le, A light, chondritic xenolith in the Murchison (CM) chondrite–Formation by fluid-assisted percolation during metasomatism? *Geochemistry* **79**, 125518–125530 (2019).

75. P. Hoppe, E. Zinner, Presolar dust grains from meteorites and their stellar sources. *J. Geophys. Res.* **105**, 10371–10385 (2000).
